# The Three-Dimensional Structure of the Genome of the Dark Septate Endophyte *Exophiala tremulae* and Its Symbiosis Effect on Alpine Meadow Plant Growth

**DOI:** 10.3390/jof11040246

**Published:** 2025-03-24

**Authors:** Chu Wu, Junjie Fan, Die Hu, Honggang Sun, Guangxin Lu, Yun Wang, Yujie Yang

**Affiliations:** 1 College of Horticulture & Gardening, Yangtze University, Jingzhou 434025, China; wuchu08@yangtzeu.edu.cn (C.W.); yjyang@yangtzeu.edu.cn (Y.Y.); 2 College of Life Science, Yangtze University, Jingzhou 434025, China; 19803600296@163.com (J.F.); 1wangyun@yangtzeu.edu.cn (Y.W.); 3 Research Institute of Subtropical Forestry, Chinese Academy of Forestry, Hangzhou 311400, China; honggangsun@caf.ac.cn; 4 College of Agriculture & Animal Husbandry, Qinghai University, Xining 810016, China; lugx74@163.com

**Keywords:** 3D genome structure, topologically associating domains, chromatin loops, chromosome compartments, contraction and expansion gene family, alpine meadow plants

## Abstract

The establishment of artificial grassland is a good pathway for resolving serious social and economic problems in the Qinghai–Tibet Plateau. Some beneficial indigenous microbes may be used to improve productivity in artificial grassland. The genome of the indigenous dark septate fungus, *Exophiala tremulae* CICC2537, was sequenced and assembled at the chromosome level using the PacBio sequencing platform, with the assistance of the Hi-C technique for scaffolding, and its 3D genome structures were investigated. The genome size of *E. tremulae* is 51.903848 Mb, and it contains eight chromosomes. A total of 12,277 protein-coding genes were predicted, and 11,932 genes (97.19%) were annotated. As for the distribution of exon and intron number and the distribution of gene GC and CDS GC, *E. tremulae* showed similar distribution patterns to the other investigated members of the genus *Exophiala*. The analysis of carbohydrate-active enzymes showed that *E. tremulae* possesses the greatest number of enzymes with auxiliary activities and the lowest number of enzymes with carbohydrate-binding modules among the investigated fungi. The total number of candidate effector proteins was 3337, out of which cytoplasmic and apoplastic effector proteins made up 3100 and 163, respectively. The whole genome of *E. tremulae* contained 40 compartment As and 76 compartment Bs, and there was no significant difference in GC content in its compartment As and Bs. The whole genome of *E. tremulae* was predicted to contain 155 topologically associating domains (TADs), and their average length was 250,000 bp, but there were no significant differences in the numbers of genes and the GC content per bin localized within the boundaries and interiors of TADs. Comparative genome analysis showed that *E. tremulae* diverged from *Exophiala mesophila* about 34.1 (30.0–39.1) Myr ago, and from *Exophiala calicioides* about 85.6 (76.1–90.6) Myr ago. Compared with all the investigated fungi, the numbers of contraction and expansion gene families in the *E. tremulae* genome were 13 and 89, respectively, and the numbers of contraction and expansion genes were 14 and 670, respectively. Our work provides a basis for the use of the dark septate fungus in alpine artificial grassland and further research into its symbiosis mechanisms, which may improve the growth of plant species used in the Qinghai–Tibet Plateau.

## 1. Introduction

Endophytic fungi can colonize different plant organs, such as leaves, roots, and rhizomes, but they do not cause obvious symptoms in these colonized plant organs. The main characteristic of these endophytic fungi is that they reside and grow within plant organs, and sporulate during host senescence [1]. Endophytic fungi were divided into two major groups, i.e., clavicipitaceous and non-clavicipitaceous endophytes. The former only includes class 1 endophytes, while the latter includes class 2, 3, and 4 endophytes [1]. Dark septate endophytes (DSEs) belong to class 4. DSEs show two major characteristics, i.e., melanized and septate hyphae. In various ecosystems, DSEs can colonize more than 600 plant species, 320 genera, and 114 families, including plant species in Dicotyledoneae, Monocotyledonae, Gymnospermae, Equisetopsida, Lycopsida, Polypodiopsida, and Psilotopsida [2,3,4]. Just like other beneficial microorganisms, such as arbuscular mycorrhizal fungi, ectomycorrhizal fungi, root endophytic fungi, and plant growth-promoting rhizobacteria, DSEs endow their hosts with benefits [5,6], especially under heavy metal [7,8,9], drought [10,11], salt [12,13,14,15], and biotic stress [16,17,18]. In addition, DSEs possess the ability to dissolve phosphate to improve growth in their host plants [19], and they often coexist with different mycorrhizal fungi [3]. This coexistence can synergistically enhance the cadmium tolerance of their host plants [20]. The pervasive associations between DSEs and tree roots and soil microbiomes suggest the important roles of DSEs in these ecosystems [21].

In the face of the challenges brought about by climate change, DSEs show their importance in agriculture and forestry [6]. The alpine meadow in the Qinghai–Tibet Plateau is important for society and the economy in the area, and is an ecosystem sensitive to climate change. The ecosystem is facing two increasingly serious challenges, i.e., global climate change and population increase. In this case, the establishment of artificial grassland is important for providing enough forage for livestock in this area. Therefore, the question of how to improve productivity in artificial alpine grassland is essential for maintaining great quantities of local livestock and improving the economic status of local herdsmen. In view of the multiple benefits of symbiosing microorganisms in the Qinghai–Tibet Plateau [22,23,24,25], some of them may be used to increase biomass accumulation inartificial alpine grassland. A dark septate fungus, *Exophiala tremulae*, was isolated from *Cordyceps sinensis* in the Qinghai–Tibet Plateau, a famous Chinese herbal medicine (http://m.china-cicc.org/cicc/detail2/?sid=7697, accessible on 15 December 2024), but its effects on some of the main plant species used in the alpine artificial grassland in the Qinghai–Tibet Plateau are still unclear.

The genus *Exophiala* belongs to Herpotrichiellaceae, Chaetothyriales, and Chaetothyriomycetidae. Most members in the genus cause various diseases in animals and humans, such as *Exophiala dermatitidis* [26,27,28], *Exophiala angulospora* [29], and *Exophiala lecanii-corni* [30]. However, some members of the genus seem to play an important role in plant nutritional cycles. For example, *Exophiala quercina* was found on dead wood of *Quercus* sp. [31], *Exophiala eucalypticola* and *Exophial aeucalyptorum* on leaf litter of *Eucalyptus* species [32,33], and *Exophiala italica* on dead branches of *Cytisus scoparius* [34]. *Exophiala embothrii* was isolated and identified from the rhizosphere of *Embothrium coccineum* in Chile [35], but its effects on plants are not clear. Furthermore, few members of the genus *Exopiala* can symbiose with plant species in roots, such as *Exophiala pisciphila* [8,36], *Exophiala* sp. LHL08 [37], *Exophiala radicis* [38], and *Exophiala spartinae* [39]. *Exophiala tremulae* can colonize roots of *Populus tremuloides* [38,40], and the fungus was named after its host species. Similarly, these DSEs can improve plant growth and tolerance to abiotic stresses [8,36,37,41].

Some high-throughput chromatin conformation capturing technologies, such as Hi-C, ChIA-PET, and HiChIP, have been used to investigate the spatial characteristics and functions of plant genome architecture, and it was found that the eukaryotic genome has a hierarchical 3D chromatin organization in a cell nucleus [42,43]. Increasing evidence suggests that 3D genome architecture plays important roles in DNA replication, DNA repair, and gene expression regulation [23,42,43,44,45]. At present, some 3D genome structures of microbes have been investigated [43,46,47]. Naturally, DSEs have their own 3D genome architectures. In particular, we should pay attention to the changes in their 3D genome architectures during symbiosis with plant hosts, and the expression regulation of important genes involved in symbiosis processes and effects on host plants.

At present, few members in the genus *Exophiala* have been sequenced (https://www.ncbi.nlm.nih.gov/datasets/genome/?taxon=5583, accessible on 15 December 2024), but none of them can symbiose with plant roots. In this present research, we sequenced and assembled the genome of *Exophiala tremulae* at the chromosome level, analyzed its 3D genome architecture, and finally investigated its effects on plant growth after colonization in roots of *Medicago sativa*, one of the main plant species used in the alpine artificial grassland in the Qinghai–Tibet Plateau.

## 2. Experimental Materials and Methods

### 2.1. Culture and Morphological Observation of E. tremulae

*Exophiala tremulae* CICC2537 was acquired from the China Center of Industrial Culture Collection (CICC, http://m.china-cicc.org/cicc/detail2/?sid=7697, accessible on 15 December 2024). The fungus was cultured on solid PDA medium for 15 d and its hyphae were picked out for observation under a microscope. The fungus was also cultured in liquid PDA medium for 15 d and the hyphae were filtered and homogenized for 10 g FW/L. The homogenate was used to inoculate *Medicago sativa* seedlings.

### 2.2. Symbiosis Culture of E. tremulae and M. sativa Seedlings and Growth Investigation

Seeds of *Medicago sativa* cv. “Beilin 201” were provided by Prof. Guangxin Lu in Qinghai University in Xining, Qinghai province, China. The seeds were sterilized using 0.1% HgCl_2_ and sown in sterilized culture medium (peat:vermiculite = 1:1) in plastic pots (high 20 cm and diameter 15 cm). When seedlings grew to the stage with 5 true leaves, 5 seedlings were kept in the pots and others were picked out. After a week, the aforementioned hyphae homogenate was injected into culture medium in the centers of 15 plots, 10 mL for each pot. Other 15 pots were regarded as the control, and no hyphae homogenate was injected in the control pots. One month after inoculation, the growth parameters of these seedlings were investigated. Gas exchange was determined using a Li-Cor 6400 photosynthesis analysis system (Lincoln, NE, USA). Thirty plants were chosen at random from the control and the inoculated treatment for analysis of their weight, height, and total root length.

### 2.3. Extraction of DNA and RNA from the Hyphae of E. tremulae

After the culturing of *E. tremulae* in solid PDA medium for 15 d, its hyphae were originally collected. High-quality genomic DNA was extracted from hyphae using a modified CTAB method [48]. The quality and quantity of the extracted DNA were examined using a NanoDrop 2000 spectrophotometer (NanoDrop Technologies, Wilmington, DE, USA), Qubit dsDNA HS Assay Kit with a Qubit 3.0 Fluorometer (Life Technologies, Carlsbad, CA, USA), and electrophoresis was performed on a 0.8% agarose gel. Total RNA was extracted using Trizol reagent (Invitrogen, Carlsbad, CA, USA). RNA purity and integrity was monitored by use of a NanoDrop 2000 spectrophotometer (NanoDrop Technologies, Wilmington, DE, USA) and a Bioanalyzer 2100 system (Agilent Technologies, Palo Alto, CA, USA). RNA contamination was assessed by 1.5% agarose gel.

### 2.4. Construction of PacBio HiFi Library

The SMRTbell library was constructed using the SMRTbell Express Template Prep kit 2.0 (Pacific Biosciences, Menlo Park, CA, USA). Briefly, 15 μg of the genomic DNA was carried into the first enzymatic reaction to remove single-stranded overhangs followed by treatment with repair enzymes to repair any damage that may be present on the DNA backbone. After DNA damage repair, the ends of the double-stranded fragments were polished and subsequently tailed with an A-overhang. Ligation with T-overhang SMRTbell adapters was performed at 20 °C for 60 min. Following ligation, the SMRTbell library was purified with 1× AMPure PB beads. The size distribution and concentration of the library were assessed using the FEMTO Pulse automated pulsed-field capillary electrophoresis instrument (Agilent Technologies, Wilmington, DE, USA) and the Qubit 3.0 Fluorometer (Life Technologies, Carlsbad, CA, USA). Following library characterization, 3 μg was subjected to a size selection step using the BluePippin system (Sage Science, Beverly, MA, USA) to remove SMRTbells ≤ 15 kb. After size selection, the library was purified with 1× AMPure PB beads. Library size and quantity were assessed using the FEMTO Pulse and the Qubit dsDNA HS reagents Assay kit. Sequencing primer and Sequel II DNA Polymerase were annealed and bound, respectively, to the final SMRTbell library. The library was loaded at an on-plate concentration of 120 pM using diffusion loadings. SMRT sequencing was performed using a single 8M SMRT Cell on the Sequel II System with Sequel II Sequencing Kit, and with 1800 min movies, by Frasergen Bioinformatics Co., Ltd. (Wuhan, China).

### 2.5. Construction of Hi-C Libraries

Hi-C libraries were constructed according to a previous study [49]. Briefly, samples were cross-linked under vacuum infiltration for 30 min with 3% formaldehyde at 4 °C and quenched with 0.375 M final concentration glycine for 5 min. The cross-linked samples were subsequently lysed. Endogenous nucleases were inactivated with 0.3% SDS, then chromatin DNA was digested using 100 U MboI (NEB, Ipswich, MA, USA), marked with biotin-14-dCTP (Invitrogen), and then ligated by 50U T4 DNA ligase (NEB). After reversing the cross-links, the ligated DNA was extracted by use of a QIAamp DNA Mini Kit (Qiagen, Germantown, MD, USA) according to the manufacturers’ instructions. Purified DNA was sheared to 300 to 500 bp fragments and these were further blunt-end repaired, A-tailed and adaptor-added, followed by purification through biotin–streptavidin-mediated pull-down and PCR amplification. Finally, the Hi-C libraries were quantified and sequenced on the MGI-seq platform (BGI, Shenzhen China).

### 2.6. Genome Assembly with HiFi Reads

With Three SMRT cells in the PacBio Revio platform, we generated 4.73 Gb (89.74× of the genome) highly accurate (>99%) HiFi reads. All HiFi reads data was used for genome assembly of *E. tremulae*. The draft assembly of the genome was assembled using HiFiasm (v0.16.1) [50] with default parameters, and the gfatools (https://github.com/lh3/gfatools, accessible on 15 December 2024) was used to convert sequence graphs in the GFA to FASTA format.

### 2.7. Chromosome Assignment Using Hi-C Technology

Hi-C technology can be used to anchor contigs. For anchored contigs, data of 99.11 Gb were generated from the Hi-C library and were mapped to the *E. tremulae* preliminary assembly using Juicer (v1.6) [51] with default parameters. Paired reads mapped to different contigs were used for the Hi-C-associated scaffolding. Self-ligated, non-ligated, and other invalid reads were filtered out. We applied 3D-DNA to order and orient the clustered contigs. Then, Juicer was used to filter the sequences and cluster them, and the Juicebox was applied to adjust chromosome construction manually. We finally anchored the scaffolds on eight chromosomes. In addition, the BUSCO pipeline was used to assess the completeness and accuracy of the *E. tremulae* genome.

### 2.8. Assessment of Assembly Quality

Multiple genome assessments determined the high quality of the genome of the *E. tremulae* assembly. First, Benchmarking Universal Single-Copy Orthologue (BUSCO, v3.0.2) [52] analyses showed that 99.13% of the core conserved plant genes (1600 out of 1614 embryophyta_odb10) were complete in the genome of *E. tremulae* assembly, suggesting the high completeness of the assembled genome. Meanwhile, the reads re-mapping ratio and coverage were assessed using Illumina reads and long reads. Illumina short reads were aligned to the genome using BWA MEM (v0.7.17) [53] software with default parameters, and Minimap2 (v2.24) [54] software with parameters “-ax map-ont/map-hifi” was used to map the long reads. The challenges of assembly always come from highly complex repetitive sequences. We also assessed k-mer-based quality estimates (k = 19 bp) for the genome using Merqury pipeline (v1.3) [55] with HiFi reads resulting in a quality value (QV) score.

### 2.9. Genome Annotation

#### 2.9.1. Repeat Sequence Annotation

The repetitive sequences, including tandem repeats and TEs, were searched. First, we used Tandem Repeats Finder (TRF, v4.09.1) [56] to annotate the tandem repeats. Then, TEs were identified at both the DNA and protein levels using a combination of de novo and homology-based approaches. Two methods were combined to identify the repeat contents in our genome—the homology-based method and de novo prediction. For the homology-based analysis, we identified the known TEs within the genome of *E. tremulae* using RepeatMasker (v4.1.2) [57] with the Repbase TE library [58,59]. RepeatProteinMask (v3.16, http://www.girinst.org/repbase, accessible on 15 December 2024) searches were also conducted using the TE protein database as a query library. For de novo prediction, we constructed a de novo repeat library of the genome of *E. tremulae* using RepeatModeler (v2.0.1) [60], which can automatically execute two core de novo repeat-finding programs, namely, RECON (v1.08) [61] and RepeatScout (v1.0.5) [62], to comprehensively conduct, refine and classify consensus models of putative interspersed repeats for the genome of *E. tremulae*. Furthermore, we performed a de novo search for long terminal repeat (LTR) retrotransposons against *E. tremulae* sequences using LTR_FINDER (v1.0.7) [63]. We also identified tandem repeats using the Tandem Repeat Finder (TRF) package (v4.09.1) [56], and the noninterspersed repeat sequences, including low-complexity repeats, satellites and simple repeats, using RepeatMasker (v4.1.2) [57]. Finally, we have merged the lib library files of the two methods and used RepeatMasker (v4.1.2) [57] to identify the repeat contents.

#### 2.9.2. Gene Annotation

We used homologous, ab initio and transcriptome-assisted annotation to predict the structure of coding genes. For homologous annotation, Tblastn (v2.11.0+) [64] was used to compare the related species to the reference genome. Then, the aligned sequences and their corresponding proteins were filtered and transmitted to the Exonerate (v2.4.0) [65] for accurate alignment. Augustus (v3.4.0) [66,67,68] was used for de novo annotation. For RNA-seq data, we use both de novo and genome-based transcriptome assemblies. RNA-seq alignments were produced using HiSat2 (v.2.2.1) [69], and then RNA-seq alignments were further assembled into transcripts with genome-guided assembler Stringtie (v.2.1.7) [70]. Additionally, the transcriptome was assembled de novo using Trinity (v2.8.5) [71]. We built a comprehensive transcriptome database using all transcripts from RNA-seq according to the PASA pipeline (v2.4.1) [72]. Maker (v3.01.03) [73] was used to integrate the predicted gene sets into a nonredundant, more complete and reliable gene set. Finally, the PASA pipeline (v2.4.1) [72] was used to update maker consensus predictions, adding models for alternatively spliced isoforms.

#### 2.9.3. Functional Annotations

Gene functions were inferred according to the best match of the alignments to the National Center for Biotechnology Information (NCBI), Non-Redundant (NR), Kyoto Encyclopedia of Genes and Genomes (KEGG) database [74], Gene Ontology (GO) [75], TrEMBL [76] and Swiss-Prot [76] protein databases using Diamond BLASTP (v2.0.7) [77] with an E-value threshold of 1 × 10^−5^. The protein domains were annotated using InterProScan (v5.50-84.0) [78] based on InterPro [79] protein databases (https://www.ebi.ac.uk/interpro/, accessible on 15 December 2024).

#### 2.9.4. Annotation of Non-Coding RNA Genes

We used tRNAscan-SE (v2.0.9) algorithms [80] with default parameters to identify the genes associated with tRNA, which is an adaptor molecule composed of RNA used in biology to bridge the three-letter genetic code in messenger RNA (mRNA) with the twenty-letter code of amino acids in proteins. RNAmmer (v1.2) [81] was used to predict rRNA sequences. snoRNAs are a class of small RNA molecules that guide the chemical modifications of other RNAs, mainly ribosomal RNAs, and transfer RNAs and small nuclear RNAs. MiRNAs and snRNAs were identified by Infernal (v1.1.2) [82] software against the Rfam (v14.6) database [83] with default parameters.

### 2.10. Comparative Genome Analysis

#### 2.10.1. Gene Family Identification

To cluster families from protein-coding genes, we used proteins from the longest transcripts of every gene from *Exophiala tremulae* and other fungal species, i.e., *Saccharomyces mikatae*, *Candida glabrata*, *Eremothecium gossypii*, *Trichoderma reesei*, *Phytophthora sojae*, *Phytophthora ramorum*, *Schizosaccharomyces pombe*, *Laccaria bicolor*, *Serendipita indica*, *Saccharomyces cerevisiae*, *Candida albicans*, *Aspergillus niger*, *Exophiala calicioides*, *Exophiala spinifera* and *Exophiala mesophila*. We filtered alternative splicing for each gene and retained only the longest transcript of each gene to represent the coding region. Then the protein-coding genes with an ostensibly complete CDS were retained. Ostensibly complete refers to the CDS, as derived exclusively from the assembly, which starts on a codon boundary with a start codon, ends on a codon boundary with a stop codon, and has no internal stop codons. Protein sequences from these species were used to perform gene family construction using OrthoFinder2 (https://github.com/davidemms/OrthoFinder-Dockerfile/, accessible on 15 December 2024) [84,85].

#### 2.10.2. Phylogenetic Analysis

To reveal phylogenetic relationships among *Exophiala tremulae* and the above-mentioned fungal species, protein sequences from 186 single-copy ortholog genes were used for phylogenetic tree reconstruction. The protein sequences of the single-copy ortholog genes were aligned with MUSCLE (v3.8.31) program [86], and the corresponding Coding DNA Sequences (CDS) alignments were generated and concatenated with the guidance of protein alignment. RAxML (v8.2.11) [87] was used to construct the phylogenetic tree with the maximum Likelihood method. The phylogenetic relationship of other closely related species was consistent with previous studies.

#### 2.10.3. Gene Family Expansion and Contraction Analysis

According to divergence times and phylogenetic relationships, 173 and 338 gene families were significantly expanded and contracted, respectively, in the genome of *E. tremulae* (*p* < 0.05). Based on the identified gene families and the constructed phylogenetic tree with the predicted divergence times of those fungal species, we used CAFE (v4.2.1) [88] to analyze gene family expansion and contraction. In CAFE, a random birth and death model was proposed to study gene gain or loss in gene families across a specified phylogenetic tree. Then, a conditional *p*-value was calculated for every gene family, and a family with a conditional *p*-value less than 0.05 was considered to have an accelerated rate for gene gain or loss. These expansion and contraction gene families in *E. tremulae* (*p* ≤ 0.05) were mapped to KEGG pathways for functional enrichment analysis, which was conducted using the enrichment methods. This method implemented hypergeometric test algorithms, and the *q*-value (FDR, False Discovery Rate) was calculated to adjust the *p*-value using R package (https://github.com/StoreyLab/qvalue, accessible on 15 December 2024).

#### 2.10.4. Analysis of Positively Selected Genes

Single gene families were then extracted and the protein sequences from each family were aligned using MUSCLE with the default parameters. The corresponding CDS alignments were back-translated from the corresponding protein alignments using PAL2NAL (v14, http://www.bork.embl.de/pal2nal/, accessible on 15 December 2024), and conserved CDS alignments were extracted by Gblocks and used for further positively selected genes identification. The branch-site model of CODEML in PAML (v4.10.0) was used to test for potential positively selected genes, with the *E. tremulae* set as the foreground branch and the others as background branches [89]. The null hypothesis was that the *ω* value of each site on each branch was ≤1, whereas the alternative hypothesis was that the *ω* values of particular sites on the foreground branch were >1. A likelihood ratio test was then performed—the null distribution was a 50:50 mixture of *X*^2^ distributions with 2 degree of freedom. The *p*-values calculated based on this mixture distribution were further corrected for multiple testing by conducting an FDR test with a Bonferroni correction. The positively selected genes met the requirements of a corrected *p*-value (<0.05). Significantly overrepresented GO and KEGG terms among the positively selected genes were identified using the top GO and KEGG packages in R package.

### 2.11. Statistical Analysis

All the data obtained from *M. sativa* seedlings, including their weight, height, total root length, and gas exchange parameter, were analyzed using SPSS software (17 v.), and the means were compared between control and inoculated plants based on the least significant difference (LSD) test (*p* < 0.05).

## 3. Results

### 3.1. Morphological Observation of E. tremulae and Its Symbiosis Effect on Growth of M. sativa Seedlings

**Figure 1:**
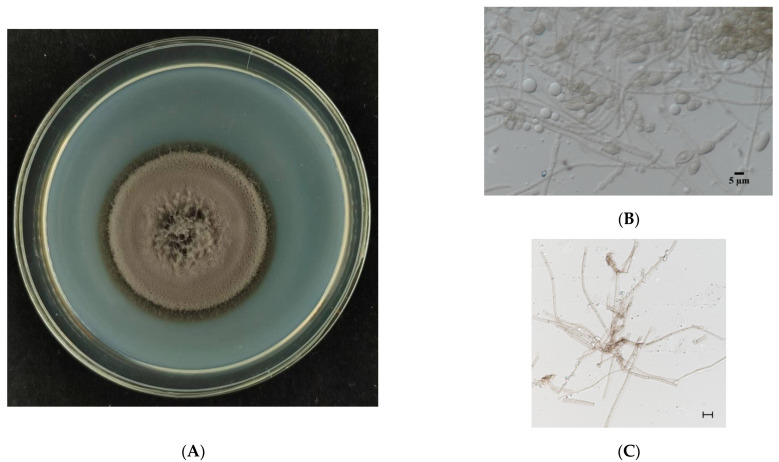

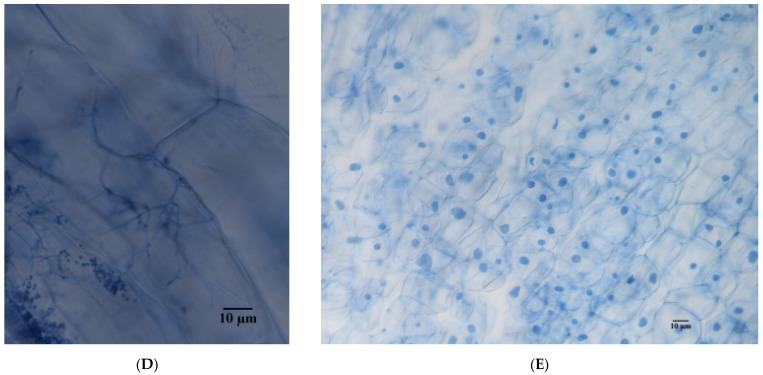
Morphology of the colony formation (**A**), the spores (**B**) and the mycelium ((**C**), bar = 10 μm) of *Exophiala tremulae* cultured in solid culture medium PDA for 15 d and its localization on the root surface (mycelium (**D**)) and in the root cortex (spores (**E**)) of *Medicago sativa* seedlings.

**Figure 2:**
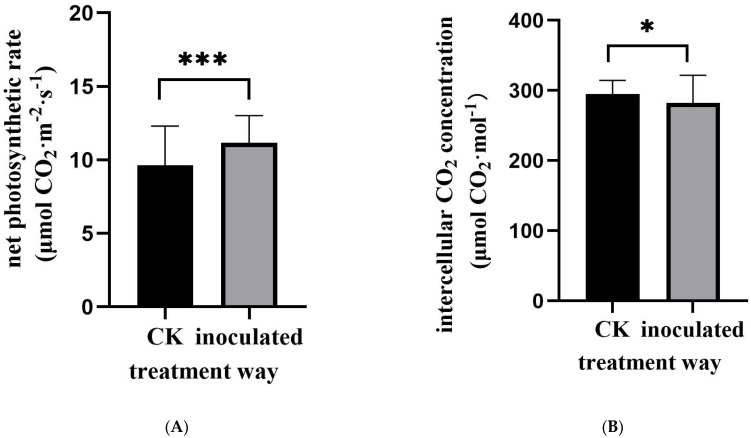

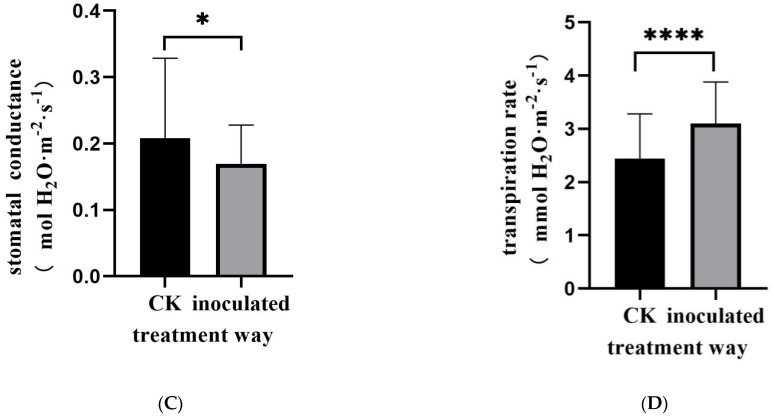
The effects of *E. tremulae* inoculation on gas exchange of *M. sativa* seedlings. (**A**) Net photosynthesis rates; (**B**) intercellular CO_2_ concentrations; (**C**) stomatal conductance; (**D**) transpiration rates. One asterisk stands for significance at *p* = 0.05; three for significance at *p* = 0.001 and four for significance at *p* = 0.0001 (mean ± SE, *n* = 30–50).

**Figure 3:**
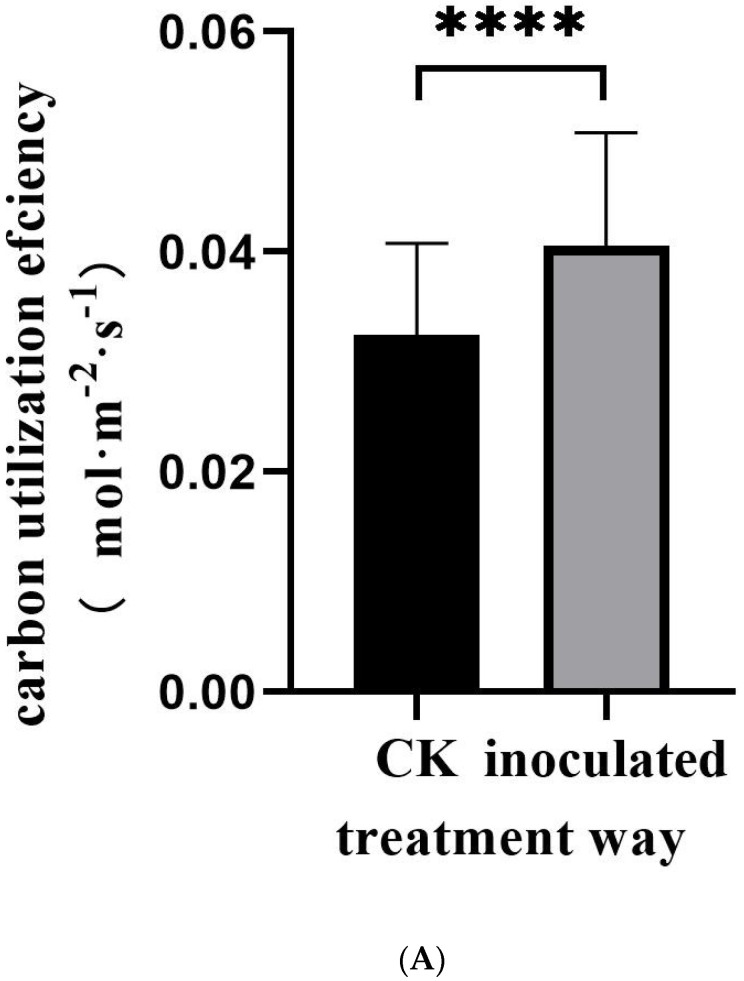

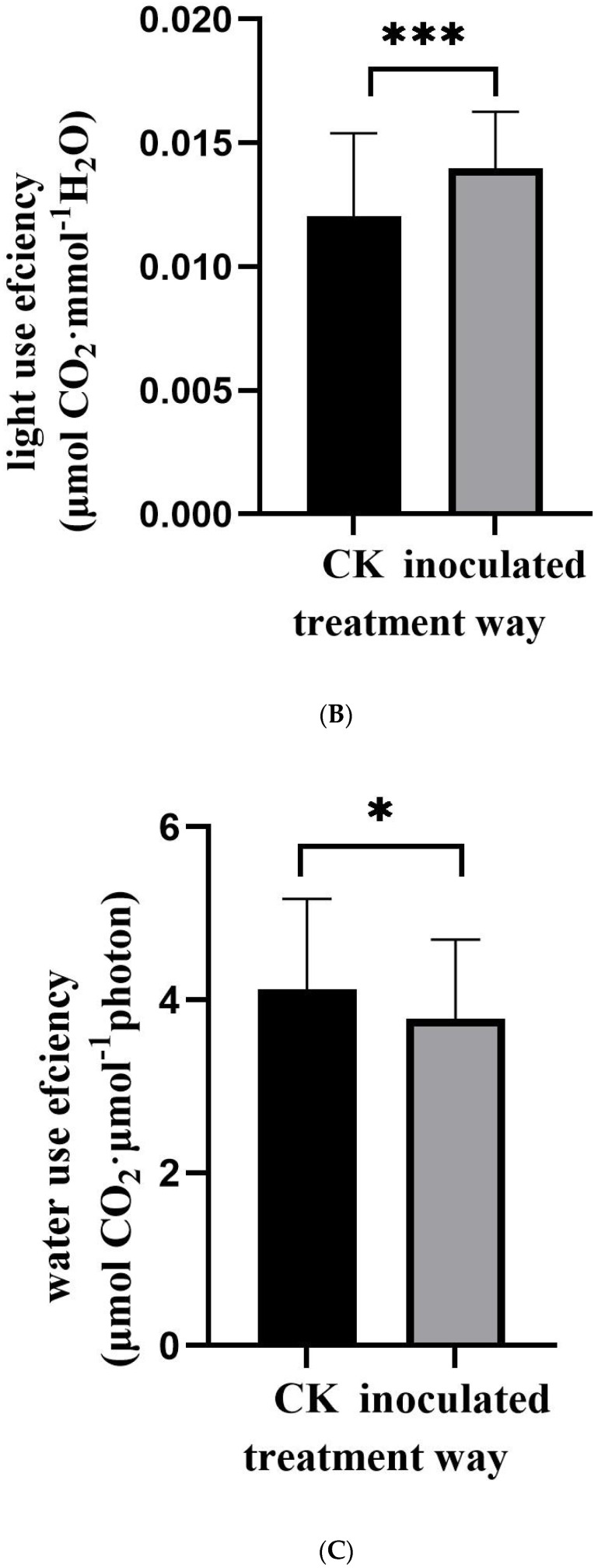
The effects of *E. tremulae* inoculation on carbon-utilization efficiency (**A**), light-use efficiency (**B**), and water-use efficiency (**C**) of *M. sativa* seedlings. One asterisk stands for significance at *p* = 0.05; three for significance at *p* = 0.001 and four for significance at *p* = 0.0001 (mean ± SE, *n* = 30–50).

**Figure 4:**
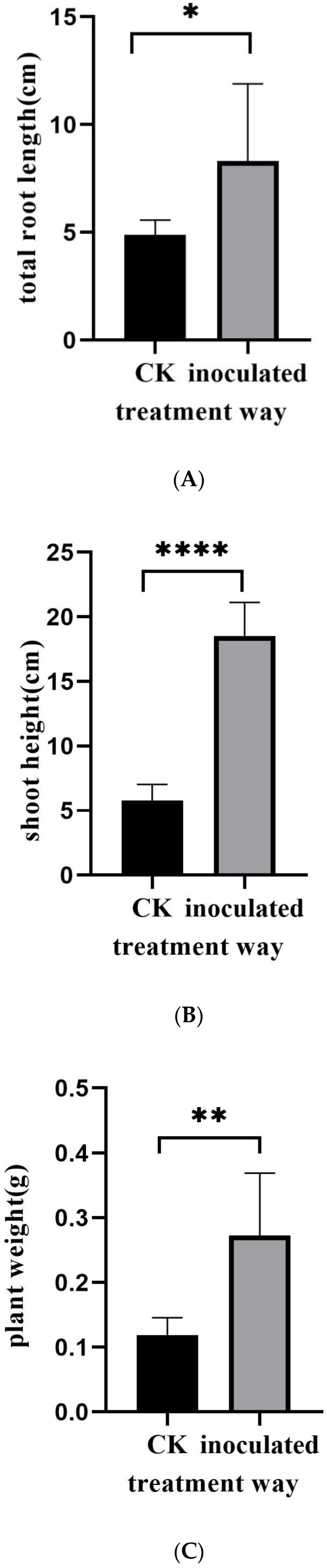
The effects of *E. tremulae* inoculation on total root length (**A**), plant height (**B**), and plant weight (**C**) of *M. sativa* seedlings. One asterisk stands for significant difference at *p* = 0.05; two for significant difference at *p* = 0.01, and four for significant difference at *p* = 0.0001 (mean ± SE, *n* = 30).

The fungus was cultured on PDA medium for 15 d (Figure 1A). Its morphology was observed (Figure 1B,C), showing that its spores were similar to those of *Exophiala spatinae* [39]. The fungus can colonize the roots of *M. satival* seedlings (Figure 1D,E). The inoculation affected the gas exchange of *M. sativa* seedlings (Figure 2). It was found that the inoculated seedlings possessed significantly higher net photosynthesis rates, intercellular CO_2_ concentrations, and stomatal conductance than the control (*p* < 0.05, Figure 2A–C), but significantly lower transpiration rates (compared with control) (*p* < 0.05, Figure 2D). Compared with the control, the inoculated seedlings showed significantly higher carbon- and light-use efficiency (*p* < 0.05, Figure 3A,B), but significantly lower water-use efficiency (*p* < 0.05, Figure 3C). Different gas exchange levels affected biomass accumulation (Figure 4). Compared with the control, the inoculated seedlings had significantly higher levels of total root length (Figure 4A), shoot height (Figure 4B), and plant weight (Figure 4C) (*p* < 0.05).

### 3.2. Chromosome-Level Assembly of the Genome of E. tremulae

The genome of *E. tremulae* was sequenced using the PacBio sequencing platform, with the assistance of the Hi-C technique for scaffolding. The length of the initial sketch of the genome sequence was 52,554,547 bp. After primary assembly, contig N50 = 6,020,537 bp, amounting to 38 contigs. After assembly with the help of the Hi-C technique, the genome length anchored to chromosomes was 51,903,848 bp, contig N50 = 4,428,737 bp and scaffold N50 = 6,147,772 bp, and 99.331% (51.903848 Mb) of the genome of *E. tremulae* was found to anchor onto 8 pseudochromosomes (Table 1, Tables S1 and S2, and the Supplementary File “Exophiala tremulae genome” in Fasta format). A Hi-C interaction map of the whole genome of *E. tremulae* with a resolution of 25 k showed eight strong interaction zones (Figure S1).

After assembly, the genome quality was evaluated. Based on the single-copy ortholog set in OrthoDB, BUSCO was used to predict the genes in the genome of *E. tremulae* and evaluate the integrity of the genome, the fragmentation extent, and the possible loss rate. Based on the evaluation, the integrity of the gene zone in the assembled result was also evaluated. In the evaluation, fungi_odb10 in BUSCO (http://www.busco.co.za/, accessible on 15 December 2024) was used, and the BUSCO evaluation results are listed in Table 2. BUSCO analysis showed that the complete BUSCOs was 99.8% (Table 1 and Table 2), suggesting a high level of the integrity of the genome. The percentages of fragmented and missing BUSCOs were 0.1% and 0.0%, respectively (Table 2). Consensus quality value (QV) was also used to evaluate the quality of the assembly. In the evaluation, QV was 64.7991 and the error rate was 3.31 × 10^−7^ (Table 1 and Table S3). Both the two evaluations suggested a high level of sequence quality of the final genome assembly. The GC percentage was 49.3%, out of which 24.66% were C and 24.64% were G (Table S4). Contig GC’s content density-sequencing depth is shown in Figure S2.

### 3.3. Genome Annotation

At first, repeat sequences were analyzed using different software methods and the contents of repeat sequences were summarized (Table S5). The total repeat size was 5,614,244 bp, accounting for 10.82% of the genome. Three analysis methods were used to analyze the repeat sequences of transposon elements, i.e., RepeatMasker, RepeatProteinMask, and de novo. After integrating the results from the three analysis methods and deleting redundancy, the total length of transposon elements was 5,423,197 bp, accounting for 10.45% of the genome (Table S5). The classification of repeat sequences of transposon elements is summarized in Table S5.

Second, the protein-encoding genes have been annotated. Six protein data banks were used to annotate the genes in the genome of *E. tremulae*, i.e., SwissProt, GO, KEGG, InterPro, NR, and TrEMBL. A total of 12,277 protein-coding genes were predicted (Table 3 and Table S6, and Supplementary files “Exophiala tremulae” in CDS format and “Exophiala tremulae genome” in GFF3 format). Of these genes, 9362 (76.26%), 9307 (75.81%), 11,324 (92.24%), 8003 (65.19%), 11,917 (97.07%), and 11,918 (97.08%) genes were annotated using InterPro, GO, KEGG, Swissprot, TrEMBL, and NR, respectively (Table 3). Out of the genes in the genome of *E. tremulae*, 345 genes (2.81%) were not annotated, and 11,932 genes (97.19%) were annotated (Table 3 and Table S7).

**Figure 5:**
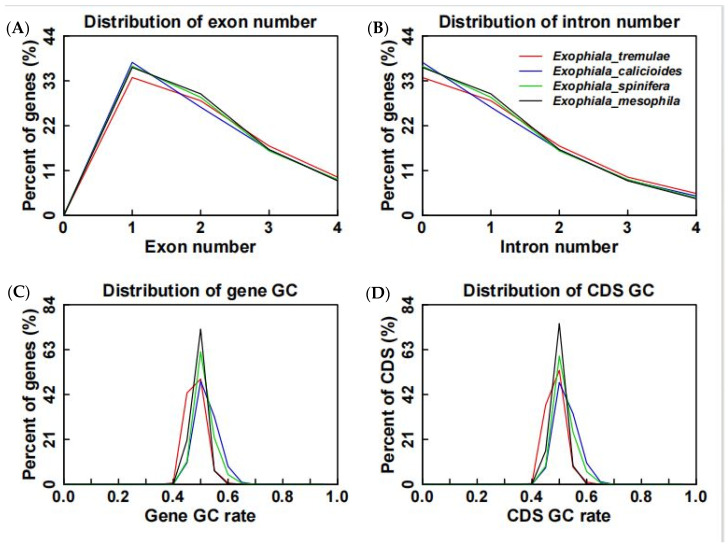
Distribution of exon numbers (**A**), intron numbers (**B**), gene GC (**C**), and CDS GC (**D**) of the four members in the *Exophiala* genus.

**Figure 6:**
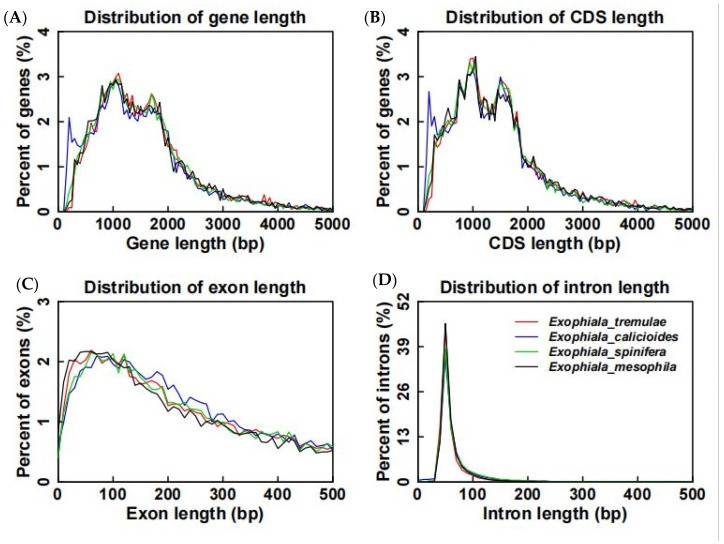
Length distributions of genes (**A**), CDS (**B**), exons (**C**), and introns (**D**) of the four members in the *Exophiala* genus.

In terms of the distribution of exon and intron numbers and the distributions of gene GC and CDS GC, *E. tremulae* showed similar distribution patterns to the other three members in the genus *Exophiala*, i.e., *E. calicioides*, *E. spinifera*, and *E. mesophila* (Figure 5)**.** At the same time, in terms of the distribution of gene length, CDS length, exon length and intron length, *E. tremulae* also showed similar patters to the three members (Figure 6). However, a comparative analysis showed that *E. tremulae* harbored the longest average gene length (1692.43 bp) and average CDS length (1564.06 bp), the highest number of average exon per gene (2.58) and the longest average exon length (605.48 bp), but the shortest average intron length (81.09 bp), among these *Exophiala* species (*E. tremulae*, *E. calicioides*, *E. spinifera*, and *E. mesophila*) (Table S8).

Furthermore, non-coding RNAs were annotated, and the results are summarized in Table S9. Interestingly, annotation analysis showed that there were no miRNA copies in *E. tremulae* (Table S9). BUSCO was used to evaluate the annotation results. The annotation results show that the annotation ratios for complete BUSCOs and for total BUSCO groups searched were 96.7% and 100%, respectively (Table S10), suggesting the high level of the annotation results.

In addition, we analyzed carbohydrate-active enzymes (CAZymes) and predicted candidate effector proteins in *E. tremulae*. Among the 16 analyzed fungi, *E. tremulae* possesses the greatest number of enzymes with auxiliary activities (AAs) and the least number of enzymes with carbohydrate-binding modules (CBMs) (Table 4 and Table S11). The fungus has more glycoside hydrolases (GHs), glycosyltransferases (GTs), and carbohydrate esterases (CEs), and less polysaccharide lyases (PLs) (Table 4 and Table S11). The genome of *E. tremulae* was predicted to contain 3337 candidate effector proteins, out of which cytoplasmic and apoplastic effector proteins were 3100 and 163, respectively (Table 5 and Table S12). Some specific effector proteins were predicted according to their own specific domains, such as CRN, RXLR, and CFEM (Table 5 and Table S12).

### 3.4. Three-Dimensional Genome Organization of E. tremulae

**Figure 7:**
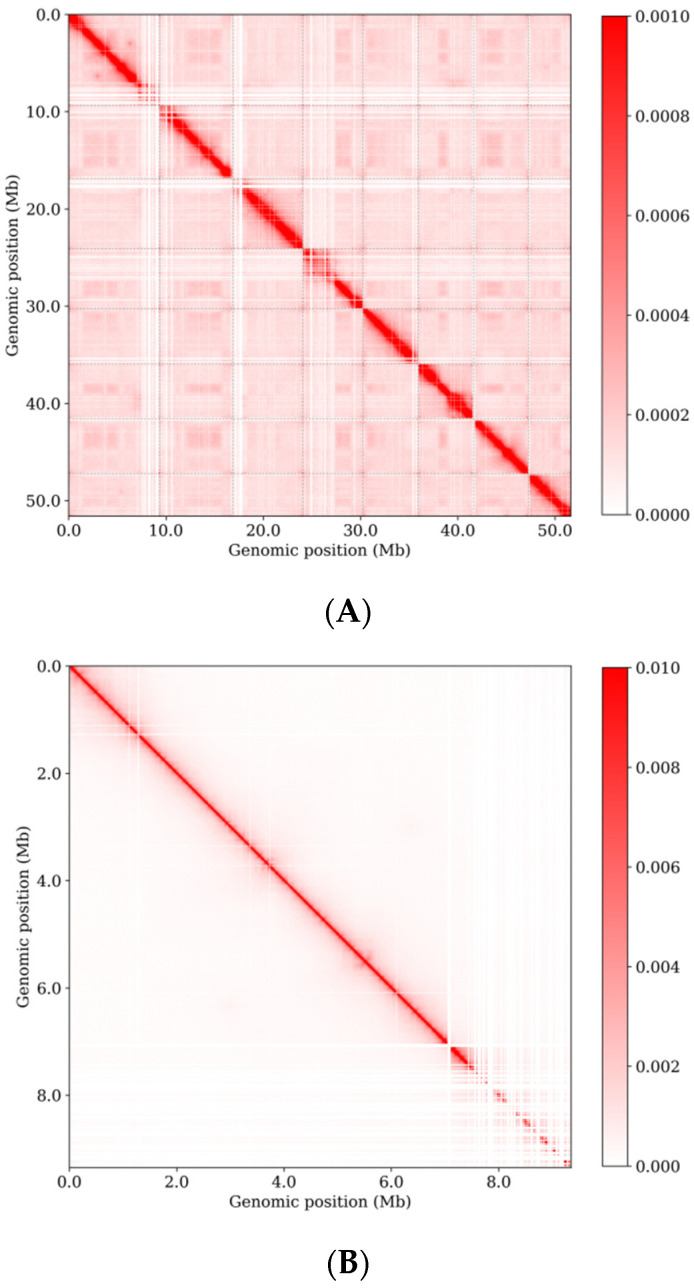

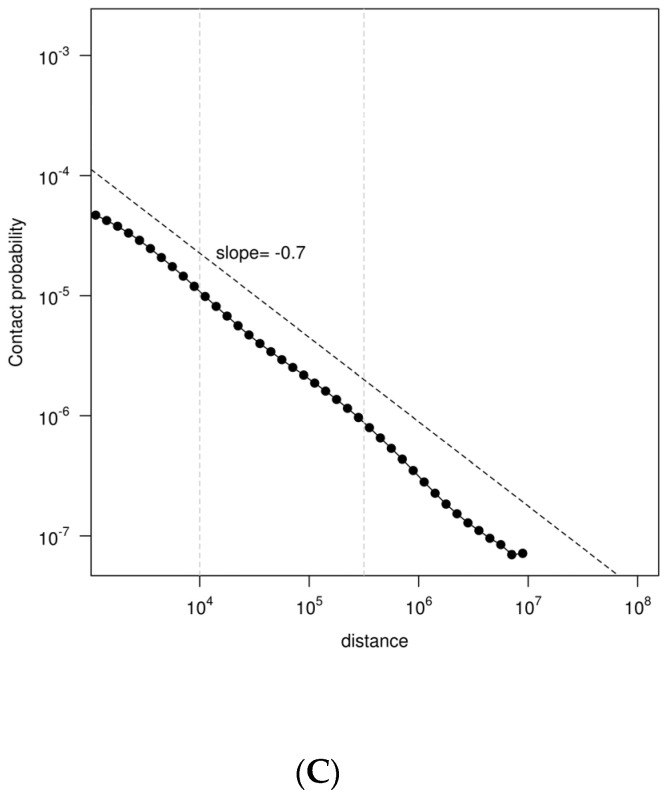
(**A**) Hi-C interactome (10-kb bins) within and among chromosomes (I to VIII). The color intensity represents the contact frequency. The more intense the color is, the more strong the interaction is. The columns beside the *x* and *y* axes indicate chromosomes, in which the blue color represents the predicted centromeres. (**B**) Heat map showing Hi-C interaction of chromosome II (1 kb min). The more intense the color is, the more strong the interaction is. (**C**) Genome-wide contact decay curve, showing the relationship between the contact probability and the distance in the whole genome of *E. tremulae*.

To understand the three-dimensional (3D) genome architecture of *E. tremulae*, the data from Hi-C were further analyzed. We generated genome-scale Hi-C interaction heat maps at resolutions of 10 kb. The heat maps revealed much more frequent intrachromosomal interactions (Figure 7B) than interchromosomal interactions (Figure 7A), suggesting that *cis*-interaction was stronger than *trans*-interaction. The contact decay curve revealed fewer long-range interactions than short-range contacts within chromosomes (Figure 7C).

**Figure 8:**
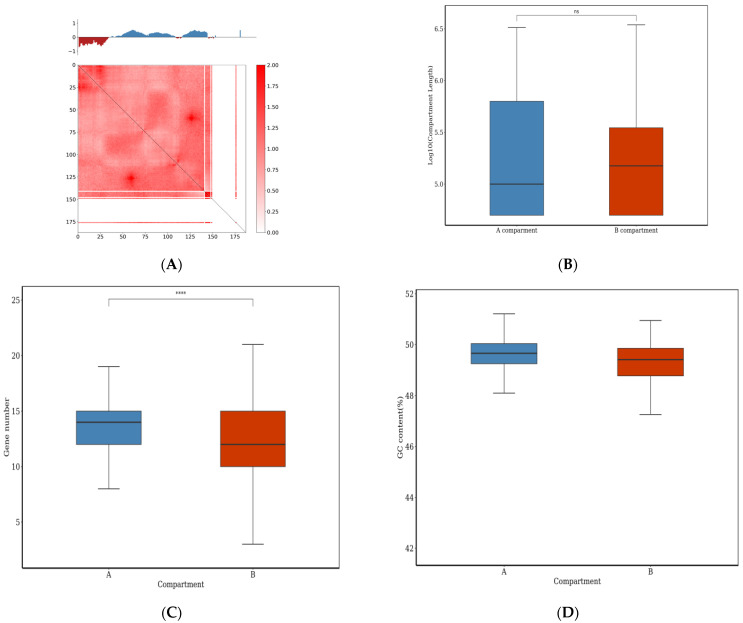
Hi-C analysis of compartment As and Bs in chromosome 1 of the genome of *E. tremulae* (**A**), length of compartment As and Bs (**B**), gene numbers in compartment As and Bs (**C**), and GC contents in compartment As and Bs (**D**). In (**A**), the upper panel shows the value distribution of compartment As and Bs in chromosome 1 of the genome of *E. tremulae*, with blue standing for compartment As and red standing for compartment Bs; the lower panel shows the heatmap of interaction in chromosome 1 of the genome of *E. tremulae*. ns stands for no significant difference; four asterisks stand for significant difference at *p* = 0.0001.

In 3D genomes, chromosome terrestrials are often divided into two parts, i.e., compartment A and B, according to their activity. The whole genome of *E. tremulae* was assembled into eight chromosomes and each of them showed different numbers of compartments (Figure S3). Herein, compartments A and B in chromosome 1 of the genome of *E. tremulae* are shown with a resolution of 50 kb (Figure 8A). In the chromosome, strong interactions occurred (low panel in Figure 8A). For the whole genome of *E. tremulae*, the numbers of compartment A and B were 40 and 76, respectively, and they respectively had 393 and 511 bins (Table S13), but their lengths were not significantly different (*p* > 0.05, Figure 8B). Compartment A is an accessible chromatin zone and is often related to euchromatin, the gene-rich zone and the active transcription zone, while compartment B is a close chromatin zone and is related to heterochromatin, gene desert, and inactive transcription zone. In compartments A and B, the numbers of genes were 5273 and 6171, respectively (Table S13), but the number per bin in compartment A was significantly higher than that in compartment B (Figure 8C).

Previous evidence shows that GC contents are dependent on cell lines, and the relationship at least in part reflects some biological significance [90], while GC contents are related to biological species, including gene density [91]. Therefore, it is of significance to link the accessible and close chromatin with GC contents. Previous research has shown that structures of compartments A and B might be related to GC contents in gene sequences [92,93]. Herein, the GC contents per bin in the whole genome of *E. tremulae* were analyzed, showing that there was no significant difference in GC contents in compartments A and B (Figure 8D and Table S13).

**Figure 9:**
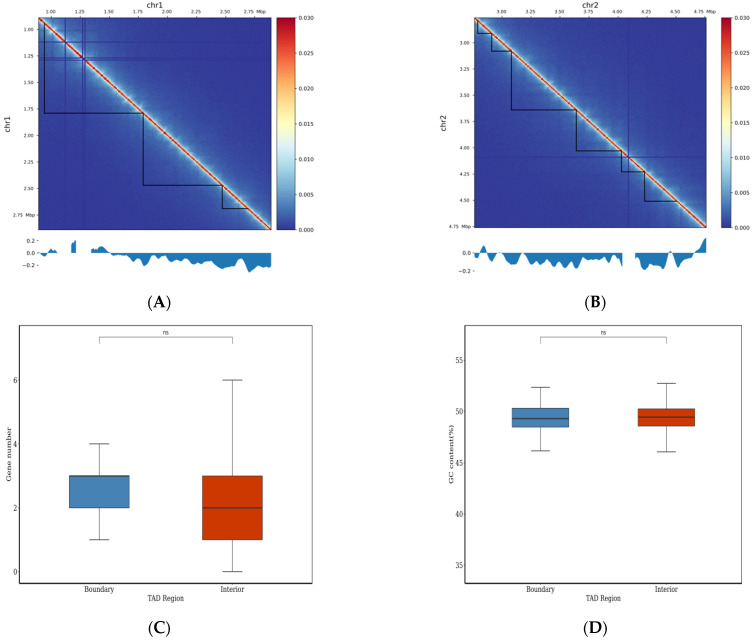
Hi-C analysis on TADs in chromosome 1 (**A**) and chromosome 2 (**B**) of the genome of *E. tremulae* with 10 kb resolution, gene numbers and GC contents in the boundary (**C**) and interior (**D**) of TDA regions of the whole genome. In (**A**,**B**), the upper panels show Hi-C heatmaps of the interaction of randomly selected zones in the chromosome; the lower panels show insulation scores of corresponding zones, and the lowest values correspond to the TAD boundaries in the chromosomes. ns stands for no significant difference.

Topologically associating domains (TADs) are a basic organization form of spatial structures of chromosomes. In general, interaction in TADs is more frequent than that occurring between two TDAs, and there are some factors in the TAD boundaries, such as transcription factors, transcription start sites, housekeeping genes, tRNA genes, and SINEs, maintaining the stability of TAD structure [94]. Herein, TADs in chromosomes are shown with 10 kb resolution (Figure 9A,B and Figure S4). For the analysis of TAD boundaries, some parts of chromosome 1 and 2 were randomly chosen and analyzed. As shown in Figure 9A,B, there were three and six TADs, respectively. There were different numbers of TADs in the chosen parts of chromosome 3–8 (Figure S4). In the whole genome of *E. tremulae*, there were 155 TADs, and their average length was 250,000 bp (Table S13).

**Figure 10:**
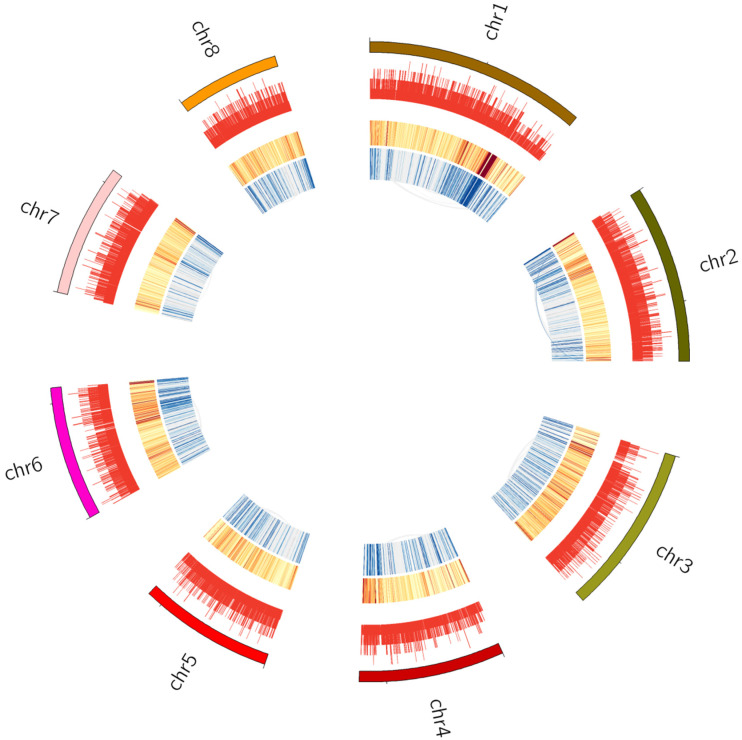
Circos diagrams indicating the genome-wide significant *cis*-interactions. The tracks (from outside to inside) indicate the chromosome names and their sites, the gene numbers, the enrichment extents of the numbers of significant interactions (red stands for a great number of *cis*-interactions at the corresponding sites), and links of significant *cis*-interaction sites (blue color intensity shows *p*-value; greater color intensities stand for smaller *p*-values).

Previous research showed that the disturbance of TAD boundaries greatly affects gene expression and results in diseases in human [95]. All bins in the whole genome of *E. tremulae* were classified into two types: localized in boundaries and in interiors of TAD. It was found that there were 148 bins localized in TAD boundaries and 5012 bins in the interior, containing 369 and 11,898 genes, respectively (Table S13), but there were no significant differences in the numbers and GC contents per bin localized in the boundaries and interiors of TADs (Figure 9C,D and Table S13). Chromatin loops often occur during gene expression because of *cis*-interactions. Herein, there were 18,876 *cis*-interaction loops in the whole genome of *E. tremulae* (Table S13), suggesting a great number of *cis*-interactions. However, there were differences in the numbers of significant interactions among the eight chromosomes of the genome of *E. tremulae* (Figure 10, chromosome assignment shown in the Supplementary fasta file).

### 3.5. Comparative Analysis on Genomes of Different Fungi

**Figure 11:**
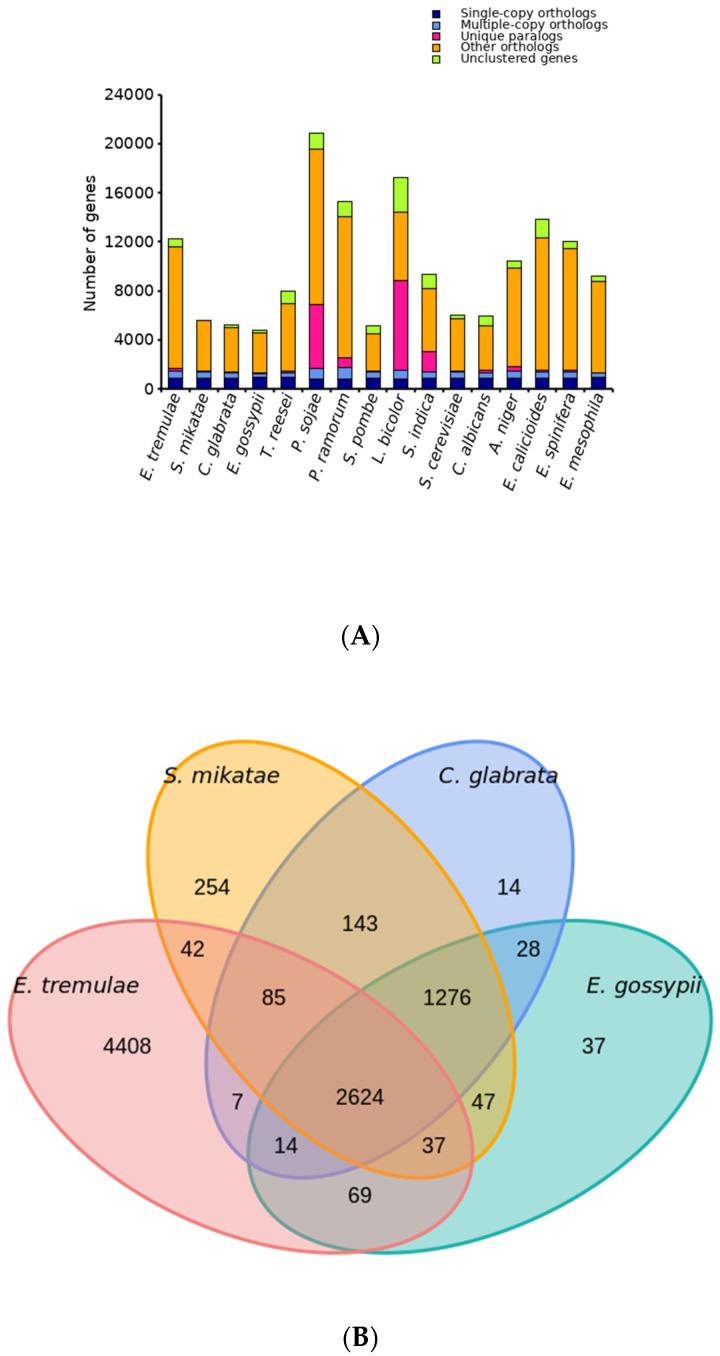
Comparison between the genomes of *E. tremulae* and other fungi. (**A**) The numbers of single-copy orthologs, multiple-copy orthologs, unique paralogs, other orthologs, and unclustered genes in the genomes of these fungal species; (**B**) Venn plot showing the unique gene families in the genome of *E. tremulae*.

At first, gene families were analyzed. We chose some microorganisms with close or distant affinities to analyze the evolution of *E. tremulae*. *E. tremulae* was predicted to possess a total number of 12,277 protein-encoding genes, more than those of *E. spinifera* and *E. mesophila*, and less than that of *E. calicioides*, but the fungus has the highest number of unique gene families among the members in the *Exophiala* genus (Figure 11A and Table S14). Compared to all the investigated fungi, the total number of genes of *E. tremulae* was higher (Figure 11A and Table S14). In the genome of *E. tremulae*, there were 7286 gene families, out of which 4408 gene families were unique, compared to those of *S. mikatae*, *C. giabrata*, and *E. gossypii*, and 2624 gene families occurred among the four species (Figure 11B and Table S14).

**Figure 12:**
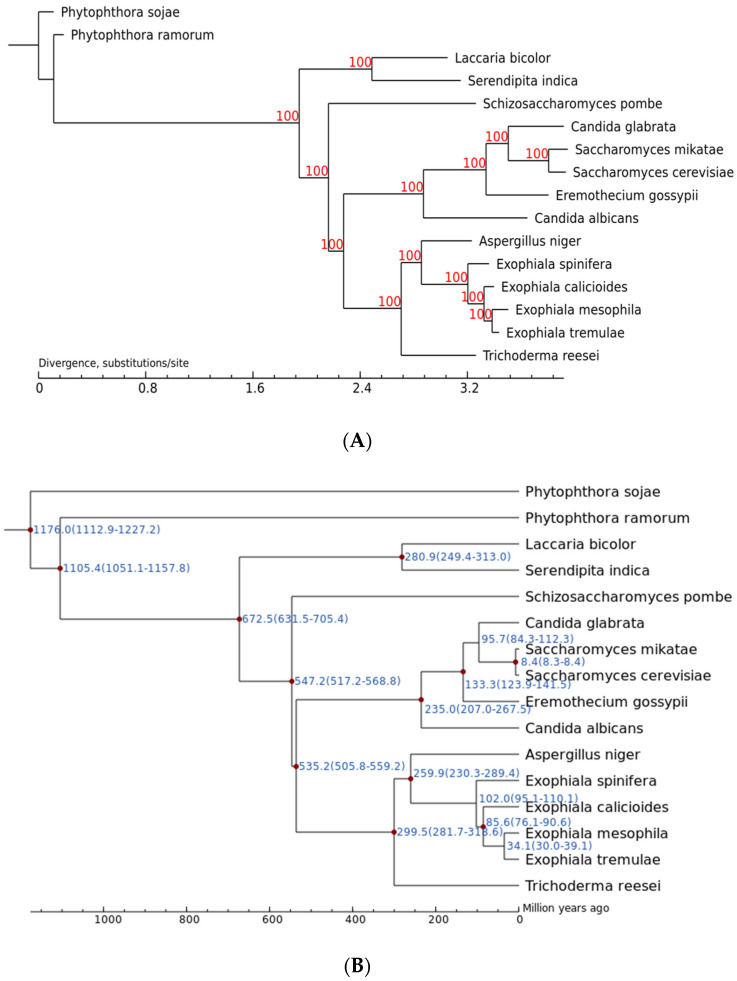
Polygenetic tree (**A**) and divergence time (**B**) of the sixteen fungi. In (**B**), the numbers at the node sites stand for the divergency times (million years) and the numbers in the parentheses stand for the fiducial intervals of the divergency times. The red nodes stand for the check values of divergency times.

Secondly, a polygenetic tree was constructed and the divergence time was estimated (Figure 12). Among the members in the *Exophiala* genus, *E. tremulae* showed closer affinity to *E. mesophila*, next to *E. calicioides* and *E. spinifera* (Figure 12A). The genus showed closer affinity to the *Aspergillus* genus and more distant affinity to the *Laccaria* and the *Serendipita* genus (Figure 12A). The analysis of divergence time showed that *E. tremulae* was diverse from *E. mesophila* about 34.1 (30.0–39.1) Myr ago, and from *E. calicioides* about 85.6 (76.1–90.6) Myr ago (Figure 12B).

Thirdly, the contraction and expansion of gene families were analyzed. Compared with all the investigated fungi, the numbers of contraction and expansion gene families of the *E. tremulae* genome were 13 and 89, respectively, and the numbers of contraction and expansion genes were 14 and 670, respectively (Table S15).

**Figure 13:**
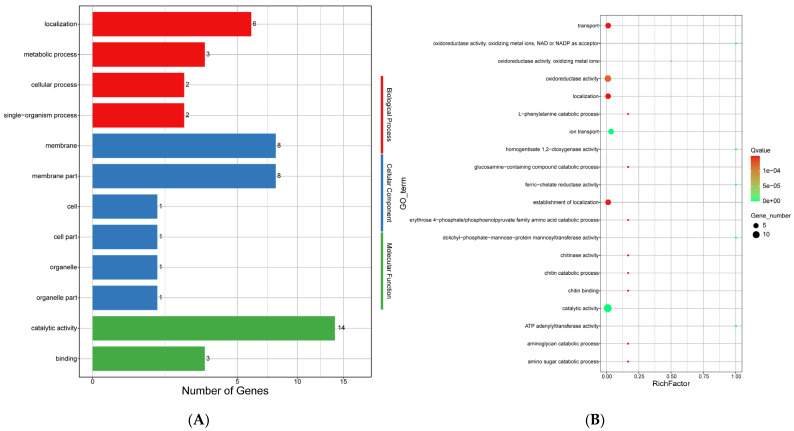

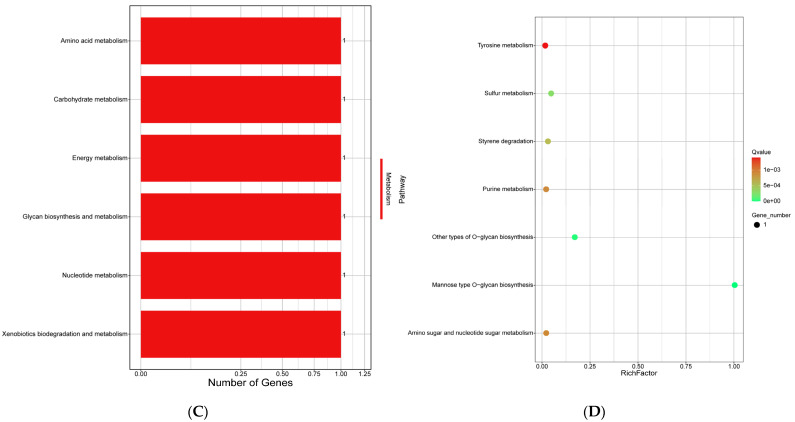
Analysis of the contraction of gene families of the genome of *E. tremulae*. (**A**) The GO term for contraction of the genome of *E. tremulae*; (**B**) the top 20 significantly enriched GO terms for contraction. (**C**) Six pathways in KEGG classification for contraction; (**D**) enriched KEGG pathways for contraction.

Comparing the genomes of *E. tremulae* and its closest relative *E. mesophila*, it was found that there were 171 and 338 gene families that expanded and contracted in the genome of *E. tremulae*, respectively, while the closest relative *E. mesophila* had 27 and 182 gene families for expansion and contraction, respectively (Figure S5). *E. calicioides* had 140 gene families for expansion and 153 gene families for contraction, while *E. spinifera* had 99 gene families for expansion and 91 gene families for contraction (Figure S5). Altogether, these results suggest that *E. tremulae* had greater numbers of gene families for expansion and contraction among the members in the *Eexophiala* genus. In the 338 gene families for contraction in the genome of *E. tremulae*, 14 genes in the GO term of molecular function had catalytic activity and 3 genes had binding function (Figure 13A). In the GO term of the cellular component, eight genes were involved in the membrane and membrane part, respectively (Figure 13A). In the GO term of the biological process, six genes were involved in localization and three genes in metabolic process (Figure 13A). Out of the top 20 significantly enriched GO terms for contraction, the main GO terms were transport, oxidoreductase activity, localization, ion transport, establishment of localization, and catalytic activity (Figure 13B). The top seven KEGG pathways for contraction were beta-alanine metabolism (33 genes), valine, leucine and isoleucine degradation (27 genes), starch and sucrose metabolism (27 genes), lysine degradation (24 genes), fatty acid degradation (23 genes), tryptophan metabolism (22 genes) and phenylpropanoid biosynthesis (20 genes) (the file “contraction_GOenrichment” in Table S16). Six pathways in KEGG classification for contraction were all involved in metabolism (Figure 13C), and the enriched KEGG pathways were mannose type *O*-glycan biosynthesis, other types of *O*-glycan biosynthesis, sulfur metabolism, styrene degradation, amino sugar and nucleotide sugar metabolism, purine metabolism, tyrosine metabolism (Figure 13D and the file “contraction_KEGGenrichment” in Table S16).

**Figure 14:**
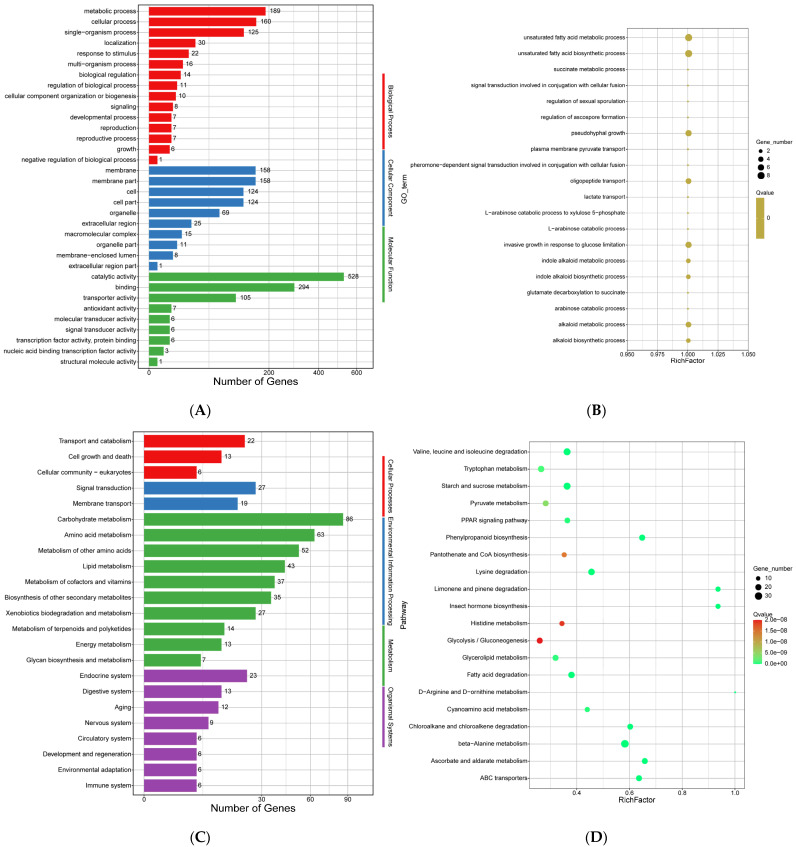
Analysis of the expansion of gene families of the genome of *E. tremulae*. (**A**) GO term for expansion of the genome of *E. tremulae*; (**B**) the top 20 significantly enriched GO terms for expansion of the genome of *E. tremulae*; (**C**) KEGG classification of the genes for expansion; (**D**) the most enriched KEGG pathways for expansion.

As shown in Figure 14A, 173 gene families showed expansion in the genome of *E. tremulae*. In the GO term of molecular function, 528, 294, and 105 genes were involved in catalytic activity, binding, and transporter activity, respectively. In the GO term of cellular component, 158, 158, 124 and 124 genes were involved in membrane, membrane part, cell, and cell part, respectively (Figure 14A). In the GO term of biological process, 189, 160, and 125 genes were involved in metabolic process, cellular process, and single-organisms process (Figure 14A). The enriched GO terms were mainly unsaturated fatty acid metabolic process, unsaturated fatty acid biosynthetic process, pseudohyphal growth, oligopeptide transport, invasive growth in response to glucose limitation, and alkaloid metabolic process (Figure 14B). The most enriched GO terms were catalytic activity (528 genes), binding (294 genes), hydrolase activity (235 genes), ion binding (235 genes), heterocyclic compound binding (224 genes), organic cyclic compound binding (224 genes), and oxidoreductase activity (201 genes) (the file “expansion_GOenrichment” in Table S16). In the KEGG classification of the genes for expansion, for organismal system, 23, 12, and 12 genes were involved in endocrine system, digestive system, and aging, respectively; for metabolism, 86, 63, and 52 genes were involved in carbohydrate metabolism, amino acid metabolism, and metabolism of other amino acids, respectively; for environmental information, 27 and 19 genes were involved in signal transduction and membrane transport, respectively; for cellular processes, 22, 13, and 6 genes were involved in transport and catabolism, cell growth and death, and cellular community, respectively (Figure 14C). The most enriched KEGG pathways were beta-alanine metabolism (33 genes), valine, leucine and isoleucine degradation (27 genes), starch and sucrose metabolism (27 genes), lysine degradation (24 genes), fatty acid degradation (23 genes), tryptophan metabolism (22 genes), and phenylpropanoid biosynthesis (20 genes) (Figure 14D and the file “expansion_KEGGenrichment” in Table S16).

**Figure 15:**
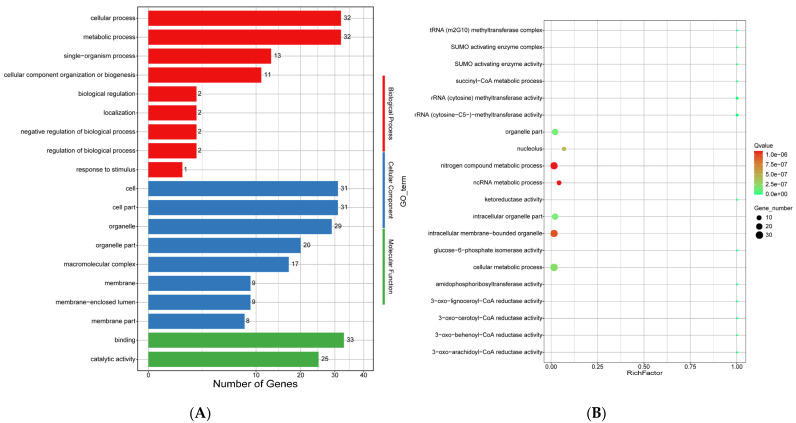

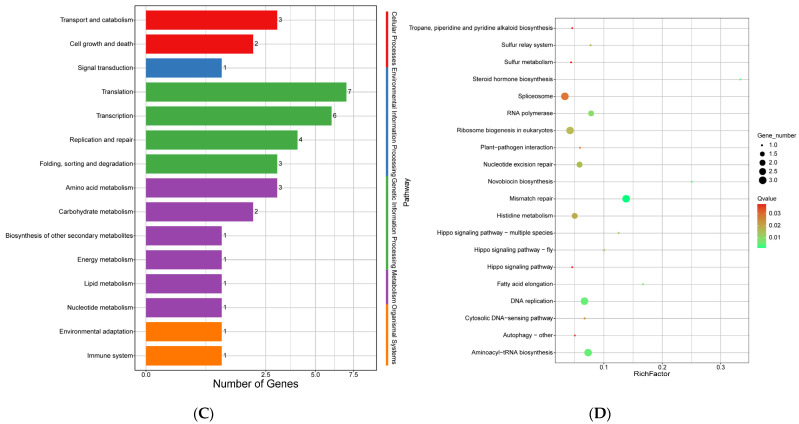
Analysis of the positive selection of genes in the genome of *E. tremulae*. (**A**) GO classification for the positive selection of genes in the genome of *E. tremulae*; (**B**) the most enriched GO terms for the positive selection of genes in the genome of *E. tremulae*; (**C**) KEGG classification for the positive selection of genes in the genome of *E. tremulae*; (**D**) KEGG enrichment analysis of positively selected genes for the positive selection of genes in the genome of *E. tremulae*.

Fourthly, the positive selection of genes in the genome of *E. tremulae* was analyzed (Table S17). GO and KEGG enrichment analyses were carried out for the genes that were positively selected. In GO classification, for molecular function, 33 and 25 genes were involved in binding and catalytic activity; for cellular component, 31, 31, and 29 genes were involved in the cell, cell part, and organelle, respectively; for biological processes, 32, 32, and 13 genes were involved in cellular process, metabolic process, and single-organism process, respectively (Figure 15A). The most enriched GO terms were organelle part, nitrogen compound metabolic process, intracellular organelle part, intracellular membrane-bounded organelle, and cellular metabolic process (Figure 15B). In KEGG classification, 20 genes were involved in genetic information processing, out of which 7 genes were in translation, 6 in transcription, 4 in replication and repair, and 3 in folding, sorting and degradation (Figure 15C). Nine genes were in involved in metabolism, out of which, three and two genes were in amino acid metabolism and carbohydrate metabolism, respectively (Figure 15C). Only one gene was involved in signal transduction (Figure 15C). KEGG enrichment analysis of the positively-selected genes showed that these genes were involved in main pathways, such as spliceosome, ribosome biogenesis in eukaryotes, mismatch repair, histidine metabolism, DNA replication, and aminoacyl-tRNA biosynthesis (Figure 15D).

## 4. Discussion

As mentioned above, most members in the genus *Exophiala* are pathogens of animals and human, and a few of them act as dark septate fungi and can symbiose with some plant species, showing benefits for their host plants. However, none of the few fungi have previously been sequenced and assembled. Their status greatly limits research on them and their application in agricultural practice. Here, we sequenced and assembled the genome of *E. tremulae* at the chromosome level (chromosome assignment shown in the Supplementary file “Exophiala tremulae genome” in FASTA format) and explored its effects on plant growth.

### 4.1. The Traits of the Genome of E. tremulae

Compared to the genomes of the other investigated members in the genus *Exophiala*, the genome of *E. tremulae* showed no traits in the distribution of exon and intron numbers, gene GC, and CDS GC (Figure 5), and no traits in the length of genes, CDSs, exons, and introns (Figure 6). However, the most significant trait of the genome of *E. tremulae* is the number of genes that encode RXLR effector proteins. RXLR effector proteins contain specific motifs, i.e., the RXLR motifs. The RXLR motif is named after the conserved Arg-X-Leu-Arg sequence located in the N-terminal regions of the effector proteins, and the motif is often followed by a dEER motif 5 to 20–25 amic acids downstream [96,97]. Microbes contain different numbers of RXLR effector proteins, such as 358 RXLR effector proteins in *Phytophthora soja* [98], 563 in *Phytophthora infestans* [99], 134 in *Hyaloperonospora arabidopsidis* [100], and at least 100 in *Plasmopara viticola* [101]. The genome of the well-studied endophytic fungus *Serendipita indica* contains 12 predicted effector proteins with the RXLRX-EER and 155 proteins with the RXXLRX-EER motifs [102]. In the genome of *E. tremulae*, it was predicted that there were 1112 genes encoding RXLR effector proteins (Table 5), suggesting that *E. tremulae* contains much more RXLR effector proteins. RXLR effector proteins show different functions during the interaction between microbes and plants [103], and are the master modulators, modifiers, and manipulators [104]. Our understanding of the functions of the RXLR effector proteins mostly stems from research on members in the genus *Phytophthora*. A great set of evidence showed that one of the functions of RXLR effector proteins is involved in their manipulation of host plant immunity. For example, the RXLR effector protein PsAvh110 in *P. sojae* targets a host transcriptional complex to modulate plant immunity [105]. The RxLR effector PcSnel4B in *Phytophthora capsici* promotes the degradation of resistance protein AtRPS2 and facilitates infection [106]. The RXLR effector protein Pi23014 in *P. infestans* targets host RNA-binding protein NbRBP3a to suppress plant immunity [107]. Their manipulation of host plant immunity facilitates their colonization in host plants. Since *E. tremulae* was predicted to possess a great number of RXLR effector proteins, some of them should facilitate its colonization in plants. Therefore, it is reasonable to speculate that *E. tremulae* can colonize a wide range of plant species, just like well-studies root endophytic fungus *S. indica* [108,109]. In view of the two facts, i.e., *E. tremulae* CICC2537 was isolated and identified from *Cordyceps inensis* in the Qinghai–Tibet Plateau (http://www.china-cicc.org/cicc/detail2/?sid=7697, accessible on 15 December 2024) and colonized in roots of *Populus tremuloides* in Canada [38,40]; the fungus can be used to inoculate plants in the areas with high altitude and altitude. Perhaps just like *S. indica* [110,111,112], the fungus promotes plant early flowering, thus reducing crop loss caused by early frosts in the areas.

Carbohydrate-active enzymes (CAZymes) are responsible for both the biosynthesis and breakdown of carbohydrates and glycoconjugates such as exopolysaccharides, starch, cellulose, and lignin, and are involved in the glycosylation of proteins and lipids [113]. CAZymes include six types of enzymes, i.e., glycoside hydrolases (GHs), glycosyltransferases (GTs), polysaccharide lyases (PLs), carbohydrate esterases (CEs), carbohydrate-binding modules (CBMs), and some enzymes with auxiliary activities (AAs) [114], and they show strong functions in the interaction between microbes and plants, because they are capable of breaking down complex polysaccharides into simpler forms and facilitate the entry of effector proteins into plant cells. *E. tremulae* was predicted to possess 82 enzymes with auxiliary activities (AAs) in CAZymes, the greatest number of AAs among the investigated microbes (Table 4). Although it was predicted that the numbers of genes encoding GHs, GTs, CEs, and CBMs in the genome of *E. tremulae* are similar to those in the investigated microbes, more genes encoding enzymes with auxiliary activities (AAs) in the genome of *E. tremulae* facilitate its colonization in plant roots and increase its effects on plant physiological activities. Knapp et al. (2018) [115] showed that *Cadophora* sp. has 150 CBMs and 12 CBMs in *Paracoccidioides brasiliensis*, the highest and the lowest numbers of CBMs among their investigated microbes, respectively. Compared to these microbes listed by Knapp et al. (2018) [115], *E. tremulae* was predicted to possess the lowest number of CBMs (5 CBMs, Table 4). At the same time, the numbers of GHs, GTs, PLs, CEs, and AAs in the predicted genome of *E. tremulae* were similar to the numbers of these microbes found by Knapp et al. (2018) [115]. Noncatalytic CBMs play important roles in the functions of lytic polysaccharide monooxygenases [23] and endoglucanases [116,117]. The main mechanism by which CBMs promote cellulase hydrolysis is to increase the accessibility of cellulose to cellulases. However, different CBMs showed diversity in affinity to cellulose substrates. For example, in the endophytic fungus *Trichoderma viride*, CBM3 showed the highest affinity for cellulose substrate, with an 84.69% adsorption rate among CBM1, CBM2, CBM3, and CBM4 [117]. Therefore, it is reasonable to speculate that *E. tremulae* has a weaker ability to degrade cellulose. In addition, just like the distribution of genes encoding cellulases in the genome of *Penicillium oxalicum* [46], the cellulase-encoding genes uniformly distributed in the genome of *E. tremulae*, such as *Etr00001.1* and *Etr00393.1* in Chromosome I, *Etr02381.1* in Chromosome II, *Etr04979.1* in Chromosome III, and *Etr05603.1* in Chromosome IV (Table S6).

### 4.2. Gene Family Expansion of the Genome of E. tremulae

The gene family expansion of the genome of *E. tremulae* showed specific traits in responding to environmental changes. The GO enrichment analysis of expansion genes showed that the genes encoding those proteins involved in the biosynthetic and metabolic processes of unsaturated fatty acids were enriched and expanded (Figure 14B). Unsaturated fatty acids are important components of biomembranes and help to maintain the fluidity of biomembranes. Under various environmental stresses, great changes in the components and amounts of unsaturated fatty acids occur in fungi. For example, in the fungus *Aspergillus fumigates*, *Δ*9-fatty acid desaturase sdeA is essential and required for unsaturated fatty acid biosynthesis, although it did not directly affect the total levels of phospholipids and sphingolipids [118]. Under high-temperature stress, fungi trigger the heat shock response controlled by heat shock transcription factors, such as HsfA, which regulates the expression of heat shock proteins. Fabri et al. (2023) [118] demonstrated that HsfA controls sdeA expression, while SdeA and Hsp 90 physically interact, suggesting that the biosynthesis of unsaturated fatty acids is related to heat shock. For bacteria, the cell membrane fluidity and fatty acid composition change in response to acid stress. For example, with a lowered pH value, the levels of saturated fatty acids decreased in the bacterium *Komagataeibacter hansenii* HDM1–3, while the level of unsaturated fatty acids was increased [119]. Under acid stress, the saturated fatty acids would decrease, manifested as significantly decreased C15:0 and C16:0; at the same time, the levels of these unsaturated fatty acids, such as octadecenoic acid, oleic acid, and cyclopropane fatty acid, significantly increased [119]. During the response to environmental stresses, the *cis*-*trans* isomerase of unsaturated fatty acids, a cytochrome-c type enzyme catalyzing the production of *trans*-unsaturated fatty acids from *cis*-unsaturated fatty acids, provides protective armor against environmental stresses for bacteria [120]. The *cis-trans* isomerization of unsaturated fatty acids triggers a decrease in the fluidity of the membrane in order to rapidly counteract the danger caused by environmental stresses. Thus, the *cis/trans* isomerization of unsaturated fatty acids has been regarded as a possible control mechanism of membrane fluidity in bacteria [121,122]. Therefore, the GO enrichment of expansion genes involving the biosynthesis and metabolism of unsaturated fatty acids suggests that *E. tremulae* would achieve strong adaptation to environmental changes. In addition, the GO classification of expansion genes showed that 158 genes, the greatest number in cellular composition, were predicted to be involved in membrane composition (Figure 14A), suggesting that the membrane composition of *E. tremulae* would be greatly different from that of those of the other investigated fungi. This great difference may help the fungus to survive in the Qinghai–Tibet Plateau.

The analysis of KEGG classification of expansion genes showed that 86 genes, the greatest number in environmental information processing, were involved in carbohydrate metabolism; the next was amino acid metabolism (63 genes) (Figure 14C). At the same time, the KEGG enrichment of expansion genes showed that starch and sucrose metabolism was enriched (Figure 14D). Out of 75 background genes, 27 genes were enriched (the file “Expansion_KEGG enrichment” in Table S16). Among these 27 enriched expansion genes, 3 genes encode trehalase (*Etr09688.1*, *Etr02154.1*, *Etr06635.1*); 4 genes encode the proteins with PA14 domain (*Etr05675.1*, *Etr10784.1*, *Etr03884.1*, *Etr11141.1*, and *Etr10685.1*); 5 genes encode the members of glycosyltransferase family 20 (*Etr02380.1*, *Etr00519.1*, *Etr05668.1*, *Etr06013.1*, and *Etr03633.1*); 10 genes encode the members of glycosyl hydrolase family (*Etr03831.1*, *Etr10195.1*, *Etr08365.1*, *Etr07272.1*, *Etr00780.1*, *Etr10753.1*, *Etr06596.1*, *Etr04231.1*, *Etr00031.1*, and *Etr06709.1*); 3 genes encode neutral trehalase with Ca^2+^ binding domain (*Etr02104.1*, *Etr04037.1*, and *Etr03566.3*); and *Etr07217.1* encodes maltase-glucoamylase (the file “Expansion_KEGG enrichment” in Table S16). All the results suggest that the fungus *E. tremulae* might possess a strong ability to use starch and sucrose. When *E. tremulae* was cultured in 2% PDA for 14 d at 22 °C, the colony diameter was only 10–15 mm [40], showing slow growth. If starch and sucrose are added into PDA, the fungus should grow faster.

The analysis of the KEGG pathway of expansion genes showed that D-arginine and D-ornithine metabolism carries out full expansion; out of five background genes, all of them (*Etr08997.1*, *Etr10351.1*, *Etr08178.1*, *Etr01194.1*, and *Etr07702.1*) are expansion genes (the file “Expansion_KEGG enrichment” in Table S16). Their expansion fortifies D-arginine and D-ornithine metabolism. At present, little is known about the significance of the increase in the function of D-arginine and D-ornithine metabolism in organisms.

In the genome of *E. tremulae*, for insect hormone biosynthesis and the degradation of limonene and pinene, the two KEGG pathways both have 15 background genes, and 14 genes out of these were expansion ones in the two pathways (the file “Expansion_KEGG enrichment” in Table S16). In the KEGG pathway of insect hormone biosynthesis, all the fourteen genes were found to encode the members of aldehyde dehydrogenase family, such as *Etr08465.1*, *Etr08291.1*, and *Etr04327.1*. The expansion genes could strengthen insect hormone biosynthesis. Maybe this is related to the isolation of the fungus *E. tremulae* from *Cordyceps inensis* in the Qinghai–Tibet Plateau (http://www.china-cicc.org/cicc/detail2/?sid=7697, accessible on 15 December 2024). *C. inensis* can infect larvae of caterpillars to generate a tonic herb that is used in traditional Chinese medicine to treat a wide range of disorders, including respiratory, kidney, liver and cardiovascular diseases, low libido and impotence, hyperlipidemia, and male reproduction [123,124]. In *E. tremulae*, insect hormone biosynthesis may be strengthened, which can help *C. inensis* to infect larvae of caterpillars. In the KEGG pathway of limonene and pinene degradation, all fourteen expansion genes encode the members of aldehyde dehydrogenase family, such as *Etr00146.1*, *Etr09722.1*, and *Etr09232.1*. The two chemicals (-)-(S)-limonene and (+)-(R)-limonene are degraded to produce many metabolites through different pathways in *Sphingomonas sanxanigenens* (https://www.kegg.jp/pathway/ssan00903, accessible on 15 December 2024). *α*-pinene is degraded to produce some metabolites (https://www.kegg.jp/pathway/map00907, accessible on 15 December 2024). Out of them, myrtenol, myrtenal, and myrtenic acid are produced through a branch pathway of *α*-pinene degradation. At present, little is known about the functions of these metabolites in association with limonene and pinene degradation in organisms.

Some genes involved in the degradation of valine, leucine, and isoleucine were enriched among the expansion genes (Figure 14D). Out of 75 background genes, 27 genes were enriched (the file “Expansion_KEGG enrichment” in Table S16). The three amino acids isoleucine, leucine, and valine are branched-chain ones. Their biosynthesis is interconnected in fungi. Different precursors are metabolized in multiple steps through shared enzymes to produce isoleucine and valine, and the valine biosynthesis pathway branches before the penultimate step to a series of leucine biosynthesis-specific steps to produce leucine [125]. However, the enrichment of genes involved in the degradation of valine, leucine, and isoleucine among expansion genes in *E. tremulae* suggests that use of the three amino acids could be strengthened when carbohydrate availability is limited, because the complete oxidation of the three amino acids in the mitochondria efficiently allows the formation of ATP by oxidative phosphorylation in plants [126]. On the other hand, the biosynthesis of valine, leucine, and isoleucine is coupled to TOR activation early in the cell cycle in yeast [127], further coordinating metabolism and cell division and determining the rate of cell proliferation. In *E. tremulae*, the KEGG enrichment of expansion genes involved in the degradation of valine, leucine, and isoleucine (Figure 14D) might be the main reason why the fungus grows slowly—10–15 mm of colony diameter when cultured in 2% PDA for 14 d [40], or 0.9–1.0 mm day^−1^ [38]. In addition, L-leucine, L-valine, and L-isoleucine are degraded to produce 3-methylbutanoly-CoA, isobutyryl-CoA, and (S)-2methylbutanoyl-CoA, respectively, and the three metabolites are the precursors of branched chain fatty acids (https://www.kegg.jp/pathway/ssan00280+NX02_02480, accessible on 15 December 2024). Just like unsaturated fatty acids, branched-chain fatty acids increase the fluidity of microbial cell membranes [128]. *Staphylococcus aureus* synthesizes branched chain fatty acids, not unsaturated fatty acids, to modulate or increase membrane fluidity, and branched chain fatty acid biosynthesis drives the tissue-specific innate immune response and infection dynamics of *S. aureus* [129]. Therefore, it reasonable to propose that the increased biosynthesis of branched chain fatty acids by the degradation of L-leucine, L-valine, and L-isoleucine is helpful for the survival and colonization of *E. tremulae*.

In the KEGG enrichment of expansion genes, tryptophan metabolism was enriched in *E. tremulae* (Figure 14D). Out of 84 background genes, 22 genes were enriched in tryptophan metabolism (the file “Expansion_KEGG enrichment” in Table S16). Among them, 14 genes encode aldehyde dehydrogenases, such as *Etr00146.1*, *Etr04631.1*, and *Etr09577.1*; 4 genes encode carbon–nitrogen hydrolases (i.e., *Etr07558.1*, *Etr04710.1*, *Etr00309.1*, and *Etr09254.1*); 2 genes, *Etr08623.1* and *Etr11051.1*, encode catalase; and the 2 genes *Etr01073.1* and *Etr02773.1* encode the proteins involved in catalase-related immune response. Aldehyde dehydrogenases are involved in tryptophan catabolism [130]. The enrichment of the expansion genes encoding aldehyde dehydrogenases promotes tryptophan metabolism, increasing the formation of some intermediates and side products involved in immune response [130].

### 4.3. Positive Selection of the E. tremulae Genome

An analysis of the positive selection of the genome of *E. tremulae* showed that highly enriched GO terms mainly included organelle part, nitrogen compound metabolic process, intracellular organelle part, intracellular membrane-bounded organelle, and cellular metabolic process (Figure 15B). In the GO subterms, there were 13 subterms in which all the numbers of background genes were hit for positive selection (the file “PGS_0.05.GO_enrichment” in Table S17). For example, in the GO subterm of the succinyl-CoA metabolic process, the gene *Etr10612.1* was hit for positive selection. The gene encodes a succinyl-CoA ligase (the file “PGS_0.05.GO_enrichment” in Table S17). Succinyl-CoA ligase (EC: 6.2.1.4, synonym: succinyl-coa synthetase) catalyzes the reaction CoA + GTP + succinate <=> GDP + phosphate + succinyl-CoA (https://www.brenda-enzymes.org/enzyme.php?ecno=6.2.1.4, accessible on 15 December 2024). Succinyl-CoA and glycine are combined by aminolevulinic acid synthase to form δ-aminolevulinic acid (dALA). dALA is an important intermediate involved in tetrapyrrole synthesis (precursor for vitamin B12, chlorophyll and heme) in vivo in microbes [131]. In the GO subterms, i.e., SUMO activating enzyme complex and SUMO activating enzyme activity, each subterm had one gene hit from one background gene for positive selection, the gene *Etr08942.1* in both the former and the latter subterm (the file “PGS_0.05.GO_enrichment” in Table S17). *Etr8942.1* encodes a protein with THIF-type NAD/FAD binding fold found in ubiquitin-activating E1 family and members of the bacterial ThiF/MoeB/HesA family, and the domain is repeated in ubiquitin-activating enzyme E1 [132,133,134]. The positive selection of SUMO activating enzyme activity in *E. tremulae* promotes protein modification by SUMO-specific activating (E1), conjugating (E2), and ligating (E3) enzymes, further regulating multiple biological processes, including cell division, DNA replication/repair, signal transduction, and cellular metabolism [135].

Forty-two KEGG pathways were enriched for positive selection (the file “PSG_0.05.KEGG_enrichment” in Table S17). Highly enriched KEGG pathways mainly included spliceosome, ribosome biogenesis in eukaryotes, mismatch repair, RNA replication, aminoacyl-tRNA biosynthesis, RNA polymerase, nucleotide excision repair, and histidine metabolism (Figure 15D and Table S17). These enriched KEGG pathways mainly included the ones involved in RNA processing (spliceosome, mismatch repair, RNA replication, aminoacyl-tRNA biosynthesis, RNA polymerase, and nucleotide excision repair) and protein biosynthesis (ribosome biogenesis in eukaryotes and histidine metabolism). The enrichment of these KEGG pathways for positive selection could strengthen the adaptation of *E. tremulae* to a changing environment and colonization in the roots of their host plants.

In the GO subterm of steroid hormone biosynthesis, one gene, *Etr10947.1*, was enriched out of three background genes (the highest hit ratio) (the file “PSG_0.05.KEGG_enrichment” in Table S17). The gene was also enriched in the GO subterm of fatty acid elongation. The gene *Etr10947.1* encodes a short chain dehydrogenase. When the filamentous fungus *Cochliobolus lunatus* was grown in a mineral medium, the yields of biomass and 17β-hydroxysteroid dehydrogenase (17/*β*-HSDH) were lower than when the fungus was grown under standard conditions, and under the former conditions, the concentration of endogenous steroids was below the limit of detection [136], suggesting that 17/*β*-HSDH activity is involved in fungal growth. Ergosterol is not only an essential structural molecule of fungal cell membranes, but also an important component of fungal growth and stress resistance [137]. Therefore, the positive selection of steroid hormone biosynthesis is helpful for the growth and stress responses of *E. tremulae*, further increasing colonization in the roots of its host plants.

In the GO subterm of novobiocin biosynthesis, one gene (*Etr11670.1*) out of four background genes was enriched for positive selection (the file “PSG_0.05.KEGG_enrichment” in Table S17). The gene *Etr11670.1* encodes aminotransferase (class I/classII). In the biosynthesis pathway of novoviocin in *Escherichia coli*, aspartate aminotransferase (EC: 2.6.1.1) is the important enzyme localized in the front of the novoviocin biosynthesis pathway, producing the important precursor of novoviocin (https://www.kegg.jp/entry/ecy00401, accessible on 15 December 2024). In *E. tremulae*, the enrichment of novobiocin biosynthesis for positive selection should improve novobiocin biosynthesis. Novobiocin is a coumarin antibiotic and is used for the inhibition of human cancer cells. For example, in SKBR3 and MCF7 human breast carcinoma cells, novobiocin interacts with heat shock protein 90 (Hsp90) and acts an antiHsp90 agent, breaking Hsp90-dependent signaling [138]. However, little is known about its functions in fungi.

In view these traits that the fungus shows in its sequence and 3D genome structures, and its symbiosis with plants in the Qinghai–Tibet Plateau, it can be used in artificial grassland in this region as well as in other fields, such as in the biosynthesis of specific secondary metabolites and the usage of specific enzymes. This research can provide a basic background for future studies on this fungus, especially those studies on gene expression regulation when it symbioses with host plants and the biosynthesis of specific secondary metabolites.

## 5. Conclusions

The dark septate endophytic fungus *Exophiala tremulae* can colonize in the roots of *Medicago sativa* and improve its growth by increasing its photosynthesis. Sequencing analysis showed that the genome size of *E. tremulae* is 51.903848 Mb, and it contains 8 chromosomes. A total of 12,277 protein-coding genes was predicted. *E. tremulae* possesses the greatest number of enzymes with auxiliary activities and the least number of enzymes with carbohydrate-binding modules among the investigated fungi. Analyses on 3D genome structures showed that the whole genome of *E. tremulae* contained 40 compartment As and 76 compartment Bs, and there was no significant difference in GC content between compartments A and B. The whole genome of *E. tremulae* was predicted to contain 155 TADs. There were strong *cis*-interactions in its eight chromosomes. Compared with all the investigated fungi, *E. tremulae* showed some specific traits of contraction and expansion gene families. Analyses of the 3D genome structures of *E. tremulae* help us understand its functions in DNA replication, DNA repair, and gene expression regulation when the fungus is under environmental stresses, or in the symbiosis processes.

## Figures and Tables

**Table 1:** Assembly statistics of the genome of *E. tremulae*.

Assembly	Test
Number of contigs	29
Assembly length (bp)	51,903,848
Contig N50 (bp)	4,428,737
anchor ratio (%)	99.331
HiFi reads mapping rate (%)	99.58
HiFi reads coverage (%)	99.96
Complete BUSCOs of genome (%)	99.8
QV	64.7991

**Table 2:** BUSCOS evaluation of the assembly sequence of the genome of *E. tremulae*.

Term	Genes	Percentage (%)
Complete BUSCOs of genome	757	99.8
Complete and single-copy BUSCOs	753	99.3
Complete and duplicated BUSCOs	4	0.5
Fragmented BUSCOs	1	0.1
Missing BUSCOs	0	0
Total BUSCO groups searched	758	100

**Table 3:** Function annotation statistics of the genome of *E. tremulae*.

	**Number**	**Percent (%)**
Total	12,277	
InterPro	9362	76.26
GO	9307	75.81
KEGG_ALL	11,324	92.24
KEGG_KO	4004	32.61
Swissprot	8003	65.19
TrEMBL	11,917	97.07
NR	11,918	97.08
Annotated	11,932	97.19
Unannotated	345	2.81

**Table 4:** Comparison of the numbers of CAZymes in different fungi.

Species	GHs	GTs	PLs	CEs	CBMs	AAs	Total
Fungi in the genus *Exophiala*							
*Exophiala tremulae* CICC2537	170	102	1	16	5	82	376
*Exophiala dermatitidis* UT8656	123	92	2	5	8	36	266
*Exophiala spinifera* CBS89968	153	114	1	8	11	42	329
*Exophiala mesophila* CBS40295	121	112	1	5	11	48	298
*Exophiala xenobiotica* CBS118157	189	119	2	9	14	68	401
*Exophiala oligosperma* CBS72588	168	137	0	10	15	65	395
*Exophiala aquamarina* CBS119918	193	113	1	8	12	52	379
*Exophiala sideris* CBS121828	146	100	0	6	14	46	312
Root endophytic fungi							
*Serendipita indica* DSM 11827	176	72	14	40	61	56	419
*Trichoderma reesei* QM6a	198	97	6	10	25	31	367
*Trichoderma harzianum* TR274	171	101	8	19	38	38	385
Arbuscular mycorrhizal fungi							
*Rhizophagus irregularis* A1 (RhiirA1_1)	32	105	9	10	17	34	207
*Gigaspora rosea* v1.0 (Gigro1)	102	196	3	23	16	60	400
Ectomycorrhizal fungi							
*Cenococcum geophilum* 1.58 (Cenge3)	171	93	2	11	30	50	357
*Lactarius deliciosus* 48 v1.0 (Lacdel1)	156	104	8	12	36	64	380
*Laccaria bicolor* v2.0 (Lacbi2)	185	97	9	18	29	61	399

Note: GHs—glycoside hydrolases; GTs—glycosyltransferases; PLs—polysaccharide lyases; CEs—carbohydrate esterases; AAs—auxiliary activities; CBMs—carbohydrate-binding modules. Except for data of *E. tremulae*, the data of the other fungi used in the table came from the website https://www.cazy.org/, accessible on 15 December 2024.

**Table 5:** The numbers of predicted effector proteins in *Exophiala tremulae*.

Types of Effector Proteins	Numbers
Total predicted effector proteins	3337
Apoplastic effector	163
Apoplastic/cytoplasmic effector	26
Cytoplasmic effector	3100
Cytoplasmic/apoplastic effector	48
CRN	172
LXAR	605
RXLR	1112
CFEM	10

## Data Availability

The original contributions presented in the study are included in the article/Supplementary Materials, further inquiries can be directed to the corresponding author.

## Supplementary Materials

The following supporting information can be downloaded at: https://www.mdpi.com/article/10.3390/jof11040246/s1, Figure S1. Hi-C heatmap showing Hi-C interaction in the whole genome of *Exophiala tremulae* at the resolution of 25 k. In this heatmap, the deeper the colors, the stronger the interactions. The X axis and Y axis stand for the positions of N*bins in the genome of *E. tremulae*. The eight squares stands for its eight chromosomes. Figure S2. Distribution of GC contents in the whole genome of *E. tremulae* (upper panel) and distribution of sequencing depth (lower panel) (1000 bp as a window). Figure S3. The whole genome of *E. tremulae* was assembled into 8 chromosomes and each of them showed different numbers of compartments. A: chromosome 1; B: chromosome 2; C: chromosome 3; D: chromosome 4; E: chromosome 5; F: chromosome 6; G: chromosome 7; H: chromosome 8. Figure S4. TADs in different chromosomes with the resolution of 10 kb. A: chromosome 3; B: chromosome 4; C: chromosome 5; D: chromosome 6; E: chromosome 7; F: chromosome 8. Figure S5. Gene expansion and contraction of the genome of *E. tremulae*. Table S1. Results after Hi-C-assisted assembly. Table S2. Chromosome characteristics of *Exophiala tremulae*. Table S3. Merqury_QV evaluation. Table S4. GC component information. Table S5. Assembly final repeat statistics. Table S6. All functional annotation. Table S7. Summary of functional analysis. Table S8. Gene structural comparison among *Exophiala* species. Table S9. ncRNA statistics. Table S10. BUSCO evaluation on functional annotation. Table S11. The numbers of CAZmyes in different fungi. Table S12. The predicted effector proteins in *Exophiala tremulae*. Table S13. Structural analysis of genomic compartments. Table S14. Statistics of different fungi about their genes. Table S15. Gene families, contraction gene families and expansion gene families. Table S16. Contraction and expansion of GO and KEGG enrichment. Table S17. Analysis results for positive selection. File S1. *Exophiala tremulae* genome (chromosome assignment) in FASTA format. File S2. *Exophiala tremulae* in CDS format. File S3. *Exophiala tremulae*. Genome. V1 in GFF3 format.
